# Glucocorticoid-mediated induction of ZBTB16 affects insulin secretion in human islets and EndoC-βH1 β-cells

**DOI:** 10.1016/j.isci.2023.106555

**Published:** 2023-04-01

**Authors:** Alexandros Karagiannopoulos, Efraim Westholm, Jones K. Ofori, Elaine Cowan, Jonathan L.S. Esguerra, Lena Eliasson

**Affiliations:** 1 Islet Cell Exocytosis, Department of Clinical Sciences-Malmö, Lund University, Malmö, Sweden; 2 Lund University Diabetes Centre, Skåne University Hospital, Lund and Malmö, Sweden; 3 Epigenetic and Diabetes, Department of Clinical Sciences-Malmö, Lund University, Malmö, Sweden; 4 Novo Nordisk A/S, Copenhagen, Denmark

**Keywords:** Endocrine system physiology, Human metabolism, Molecular mechanism of gene regulation, Bioinformatics

## Abstract

Glucocorticoid use is associated with steroid-induced diabetes mellitus and impaired pancreatic β-cell insulin secretion. Here, the glucocorticoid-mediated transcriptomic changes in human pancreatic islets and the human insulin-secreting EndoC-βH1 cells were investigated to uncover genes involved in β-cell steroid stress-response processes. Bioinformatics analysis revealed glucocorticoids to exert their effects mainly on enhancer genomic regions in collaboration with auxiliary transcription factor families including AP-1, ETS/TEAD, and FOX. Remarkably, we identified the transcription factor *ZBTB16* as a highly confident direct glucocorticoid target. Glucocorticoid-mediated induction of *ZBTB16* was time- and dose-dependent. Manipulation of *ZBTB16* expression in EndoC-βH1 cells combined with dexamethasone treatment demonstrated its protective role against glucocorticoid-induced reduction of insulin secretion and mitochondrial function impairment. In conclusion, we determine the molecular impact of glucocorticoids on human islets and insulin-secreting cells and investigate the effects of glucocorticoid targets on β-cell function. Our findings can pave the way for therapies against steroid-induced diabetes mellitus.

## Highlights

• Glucocorticoids (GCs) alter the transcriptome of human islets and EndoC-βH1 cells
• *ZBTB16* is a highly confident direct GC target in human islets and EndoC cells
• GCs negatively affect insulin secretion and strongly induce *ZBTB16* in EndoC cells
• *ZBTB16* protects EndoC cells from GC-mediated insulin secretion impairment

## Graphical abstract

**Figure:**
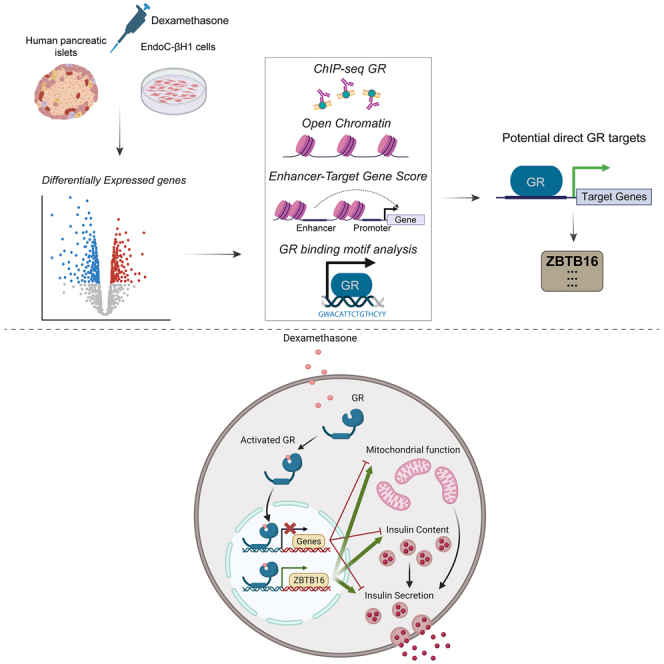

## Introduction

Glucocorticoids are steroid hormones secreted by the adrenal glands and mediate diverse immunological and metabolic effects in vertebrates.^1^ Among these, their anti-inflammatory, anti-allergic, and immunomodulatory properties are the main reason glucocorticoid analogs, such as dexamethasone, are widely prescribed in everyday clinical settings.^2^^,^^3^

Inside cells glucocorticoids act as ligands that bind to the cytosolic glucocorticoid receptor (GR), a member of the soluble nuclear receptor superfamily of ligand-dependent transcription factors,^4^ which is then activated and translocated into the nucleus. Once in the nucleus, GR binds to specific genomic elements in the vicinity of its target genes called glucocorticoid responsive elements (GREs), leading to transcriptional induction or repression of these genes.^5^^,^^6^ While GR is ubiquitously expressed in all tissues and GREs have been identified in various cell types,^7^ the glucocorticoid-responsive gene sets are cell-type specific with modest overlap between cells.^8^

Although endogenous glucocorticoids are essential in maintaining glucose homeostasis, the use of exogenous, more potent glucocorticoids used in the clinical setting has been associated with adverse metabolic effects, such as hyperglycemia and steroid-induced diabetes mellitus.^9^^,^^10^ Although steroid-induced diabetes mellitus has been mainly attributed to glucocorticoid-induced whole-body insulin resistance,^11^ growing evidence also suggest a deleterious effect of glucocorticoids on pancreatic β-cells. *In vitro* and *in vivo* studies in rodents^12^^,^^13^^,^^14^ and humans^15^^,^^16^ have demonstrated impaired β-cell function and/or reduced glucose-stimulated insulin secretion after acute and chronic glucocorticoid treatment. The consequences are more severe in susceptible individuals with reduced insulin sensitivity^17^ or glucose-stimulated insulin secretion^18^ before glucocorticoid treatment, as well as in obese females,^19^ first-degree relatives of type 2 diabetes patients^20^ and those at a higher age.^21^ It is worth mentioning that endogenous glucocorticoids such as cortisone and cortisol do not affect β-cell function and insulin secretion under physiological concentrations.^22^

In a recent study dexamethasone and glucolipotoxicity showed a synergistic negative effect on insulin secretion in EndoC-βH1 cells partly explained by an increased proinsulin/insulin ratio.^23^ Glucocorticoid-induced impairment of glucose-stimulated insulin secretion has been linked to reduced expression of genes important for β-cell function including transcription factors such as PDX-1 and NKX6-1, the exocytotic protein SYT13, and the glucocorticoid receptor GR^16^ and increased expression of glucocorticoid-regulated kinase 1 (SGK1).^16^^,^^24^ Upregulation of SGK1 increases the activity of voltage-gated K^+^ channels, which in turn reduces Ca^2+^ entry into the β-cell and thereby reduces glucose-stimulated insulin secretion.^24^ We recently showed that regulation of these proteins by glucocorticoids is in an intricate interplay with the lincRNA GAS5.^16^ ZBTB16 (zinc finger and BTB domain containing 16) is a known glucocorticoid target in non-pancreatic cell types.^25^^,^^26^^,^^27^ It has been shown to be involved in distinct biological processes such as self-renewal and differentiation of various stem cell types,^28^ limb development,^29^ spermatogenesis maintenance,^30^ and hematopoiesis.^31^ Despite being very lowly expressed in the endocrine cells of human islets,^32^ substantial induction of *ZBTB16* has recently been described in human islets after glucocorticoid treatment.^33^ However, the exact function of ZBTB16 in pancreatic islets is still not known.

Despite the characterization of important glucocorticoid gene targets in the islets and β-cells, we still have limited knowledge on the pathways regulated by glucocorticoids that lead to impaired insulin secretion. Thus, we performed differential gene expression analysis using RNA-sequencing (RNA-seq) data on both human pancreatic islets and the human β-cell line EndoC-βH1 after treatment with dexamethasone, a synthetic glucocorticoid widely used in the clinical setting. Furthermore, we integrated publicly available chromatin immunoprecipitation sequencing (ChIP-seq), chromatin state, and human enhancer data to rank our RNA-seq-defined gene targets according to their potential of being direct targets of GR. Finally, we performed functional validation and gene target identification on the top-ranked gene *ZBTB16*.

## Results

### Human islet and EndoC-βΗ1 cells display extensive transcriptomic changes after high-dose dexamethasone treatment

**Figure 1:**
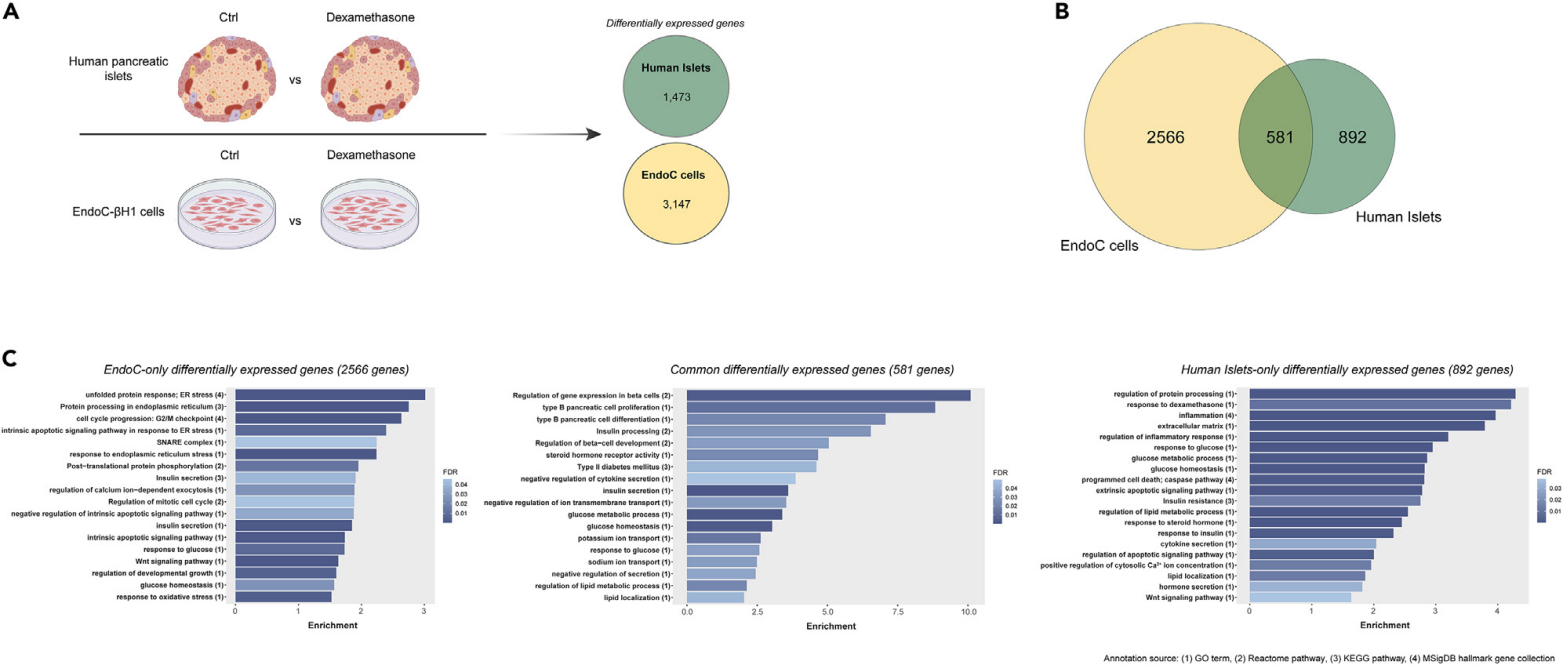
Human islet and EndoC-βΗ1 cells display extensive transcriptomic changes after high-dose dexamethasone treatment (A) Schematic representation of the RNA-seq experimental procedure after treatment with 2 μM dexamethasone. (B) Venn diagram showing the overlap of the differentially expressed genes between human islet and EndoC-βH1 cell samples. (C) Bar charts displaying selected enriched terms/pathways derived from the functional annotation of the differentially expressed genes (left) in EndoC cells (middle) in both human islets and EndoC-βH1 cells and (right) in human islets. Ctrl; Control (DMSO).

We first determined transcriptome-wide changes due to dexamethasone treatment (Figure 1A) in human islets and the human insulin-secreting cell line EndoC-βH1. EndoC-βH1 cells are widely used as translational human β-cell models due to the substantial global omics overlap with primary adult human β-cells, including their transcriptome, proteome, and secretome.^34^ Dexamethasone can affect hundreds of target genes and previous work in human islets and insulin-secreting cells have focused on key proteins in glucocorticoid signaling and β-cell function.^16^^,^^23^^,^^33^ Human islets (characteristics in Table S1) and EndoC-βH1 cells were treated for 48h and 24h, respectively, in the absence and presence of 2 μM dexamethasone before transcriptomic characterization. Analysis of the transcriptomic profiles of human islets and EndoC-βH1 cells identified 1473 and 3147 differentially expressed (DE) genes (adjusted p-value < 0.05), respectively (Figure 1A and Table S2). The overlap of DE genes between the dexamethasone-treated human islets and EndoC-βH1 cells was 581 genes (Figure 1B). Interestingly, the expression of these genes was altered in the same direction in all samples, with 309 genes being upregulated and 272 genes downregulated (Table S2). The consistency in the direction of the expression of the overlapping genes implies robust transcriptomic changes in the human β-cell and validates the results.

Functional annotation of the 581 shared DE genes revealed several enriched pathways important for β-cell function, such as glucose homeostasis, insulin processing, insulin secretion, regulation of β-cell gene expression, and β-cell proliferation and differentiation (Figure 1C, full list in Table S3). The pathway *insulin secretion* consisted of important genes for β-cell identity such as *NKX6-1* and *PDX-1*. When we instead performed functional annotation analysis of DE genes specific for either human islets (892 genes) or EndoC-βH1 cells (2566 genes) distinct genes were found to be involved in insulin secretion, glucose homeostasis, and post-translational processing. Pathways related to insulin secretion processes were more profoundly enriched in EndoC-βH1 cells. Moreover, the EndoC-βH1 DE gene set displayed a high enrichment of terms relevant to endoplasmic reticulum (ER) stress, intrinsic apoptotic signals, and cell cycle regulation, while the human islet DE gene set was enriched for terms associated with inflammation, extrinsic apoptotic pathways, response to insulin, insulin resistance, lipid metabolism, and glucocorticoid response (Figure 1C).

### Glucocorticoid Receptor regulates its targets in a distal manner and its action depends on auxiliary transcription factors

**Figure 2:**
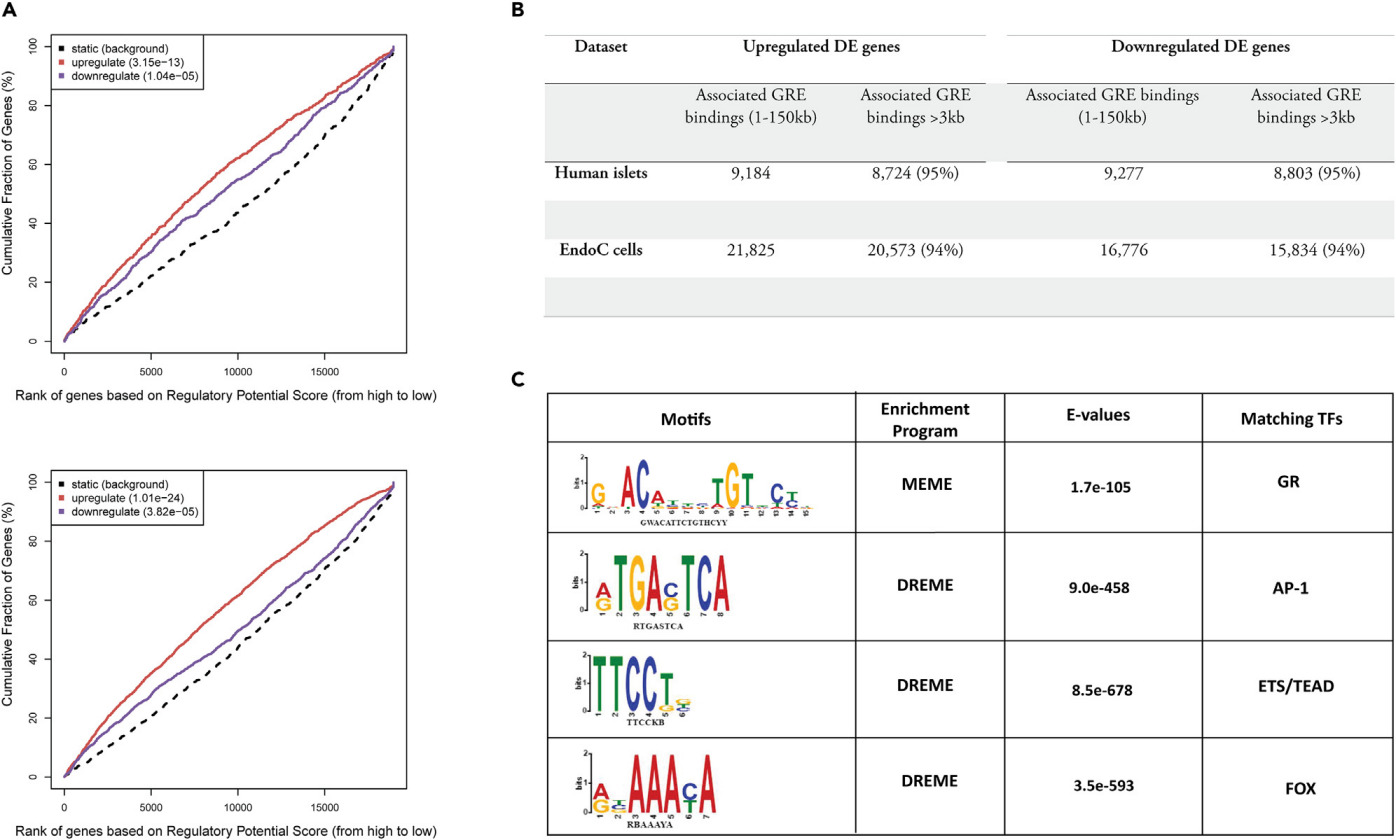
Glucocorticoid Receptor regulates its targets in a distal manner and its action depends on auxiliary transcription factors (A) Function prediction graph that represents the glucocorticoid receptor (GR) activating/repressive effect on its target genes in human islets (top) and EndoC cells (bottom) using a set of predicted glucocorticoid responsive elements (GREs). Genes are ranked based on their regulatory potential from high to low and are subsequently cumulated. The red and purple lines indicate the differentially upregulated and downregulated genes in each dataset, respectively, and the dashed line represents the non-differentially expressed genes as background. P-values measure the significance of the difference between the upregulated/downregulated and the background gene distributions as determined by the Kolmogorov-Smirnov test. (B) Number of total (positions 1 - 150000bp upstream and downstream) and distant (positions 3001-150000bp upstream and downstream) predicted GREs associated with the transcription start site of the upregulated and downregulated genes in each dataset. (C) *De novo* motif discovery in all predicted GREs with the MEME Suite. Discovered motifs, the discovery/enrichment program that generated them, and their statistical significance as represented by E-values are shown. The E-value of each motif corresponds to an estimate of the number of motifs with the same width and the number of occurrences that would have an equal or higher log-likelihood ratios if the input sequences had been generated randomly according to the background model.

We next investigated the GR binding properties of genes DE by dexamethasone in human islets and EndoC-βH1 cells. Glucocorticoids exert their effects by binding to the GR which is internalized and acts as a transcription factor. First, we obtained a high-confidence comprehensive GR genomic binding site list derived from various chromatin immunoprecipitation sequencing (ChIP-seq) experiments from the GTRD database (GR-ChIP sites).^35^ Next, we associated the obtained DE genes in islet and EndoC-βH1 sets (Figure 1) with GR-ChIP sites within a 150 kb window of their transcription start site (TSS), to yield GREs in the DE genes. After that, we calculated the estimate of the likelihood of the gene being regulated by GR using a formula that considers the distance of every associated GRE, giving a higher score with a smaller distance.^36^^,^^37^ We found that the potential of a gene to be regulated by GR was significantly higher for islet and EndoC-βH1 cell DE genes than non-DE genes, indicating that more GREs are detected closer to upregulated or downregulated DE genes than non-DE genes (Figure 2A). Moreover, the vast majority (≈95%) of GREs that were associated with the DE genes were located further from 3 kb of their TSS in both islets and EndoC-βH1 cells, suggesting that GR regulation is carried out in a distal manner (Figure 2B).

*De novo* motif discovery in the DNA sequences of all GREs revealed 4 enriched motifs that correspond to DNA binding sequences of distinct transcription factor families (Figure 2C). In addition to a motif that coincides with the GR binding sequence (GBS), motifs belonging to the AP-1, ETS/TEAD, and FOX transcription factor families were found to be enriched. Surprisingly, when GBS was scanned across GREs, only 37% of GREs displayed a stringent GBS (p < 0.0001), while 77% contained a less canonical GBS (p < 0.001). A very high proportion of GREs (95%) contained at least one of the alternative AP-1, ETS/TEAD, and FOX binding sequences besides a GBS, with the proportion rising to 97% for GREs that did not contain any canonical GBS.

### *ZBTB16* is the most strongly predicted direct glucocorticoid target in human islets and EndoC-βH1 cells

**Figure 3:**
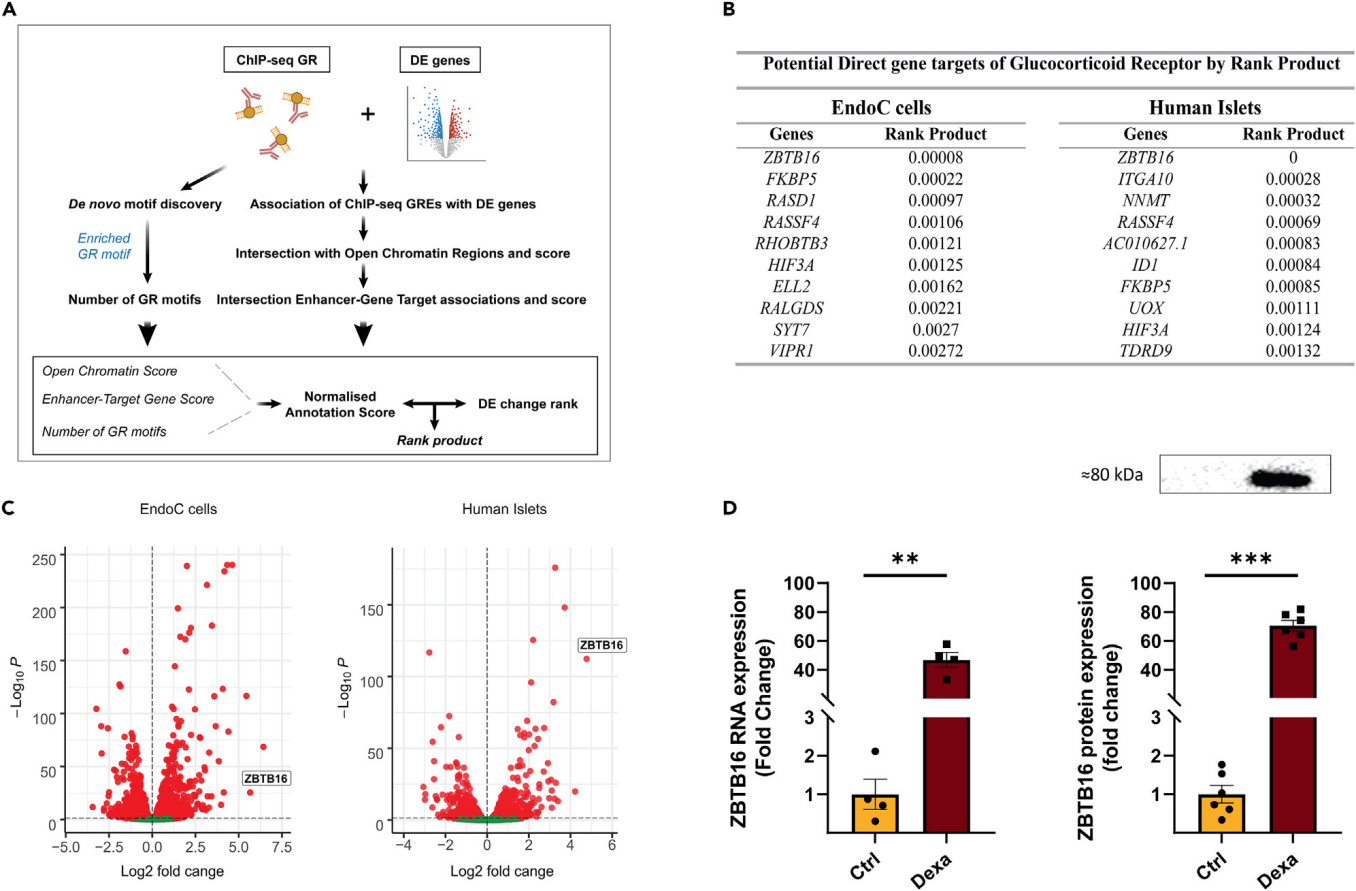
*ZBTB16* is the most strongly predicted direct glucocorticoid target in human islets and β-cells (A) Schematic summary of the bioinformatics workflow to rank differentially expressed genes according to their potential of being direct Glucocorticoid Receptor targets. (B) Top 10 differentially expressed genes upon 2 μM dexamethasone treatment with the lowest rank product. The rank product can be interpreted as a p-value, which represents the probability of the gene to be a direct target of GR. (C) Volcano plot representing differentially expressed genes upon 2 μΜ dexamethasone treatment and *ZBTB16*’s relative position compared to all genes. Significantly downregulated genes are indicated in red on the left side of the vertical dashed line and significantly upregulated genes are indicated in red on the right of the vertical dashed line. Vertical dashed lines correspond to the log2 fold change threshold of 0 separating down- and upregulation and horizontal dashed lines correspond to the adjusted p-value threshold of 0.05 represented as log_10_ adjusted p-value. (D) *ZBTB16* RNA expression (RNA-seq, left, n = 4 biological replicates) and protein expression (Western blot, right, n = 6 biological replicates) in EndoC-βΗ1 after 2 μM dexamethasone treatment. Ctrl; Control (DMSO), Dexa; Dexamethasone. Data are presented as mean ± SEM; ∗∗p < 0.01, ∗∗∗p < 0.001.

We next developed a bioinformatics pipeline (Figure 3A, described in detail in STAR Methods), to focus on potential direct glucocorticoid targets from the large list of DE genes after high-dose dexamethasone treatment. Briefly, we first associated each DE gene with GR-ChIP sites to generate GREs. Then by integrating human islet/EndoC-βH1-specific accessible chromatin region data, enhancer-target association data, and GR binding motif site information, we introduced a *Normalized Annotation Score*. By combining the *Normalized Annotation Score* with the fold-change values of each DE gene, a gene rank product was generated, which can be interpreted as a p-value that indicates the potential of the gene to be a direct glucocorticoid target. In both human islet and EndoC-βH1 cell sets, *ZBTB16* had the lowest rank product, making it the most suitable candidate to be a direct target of dexamethasone and, consequently, by GR (Figure 3B). Indeed, *ZBTB16* transcriptional induction was among the highest in human islets and EndoC-βH1 cells (Figure 3C), and both RNA and protein levels of *ZBTB16* were strongly induced upon high-dose dexamethasone treatment in EndoC-βH1 cells (Figure 3D).

### Glucocorticoid Responsive Elements are located intronically and within conserved regions of *ZBTB16* in EndoC-βH1 cells

**Figure 4:**
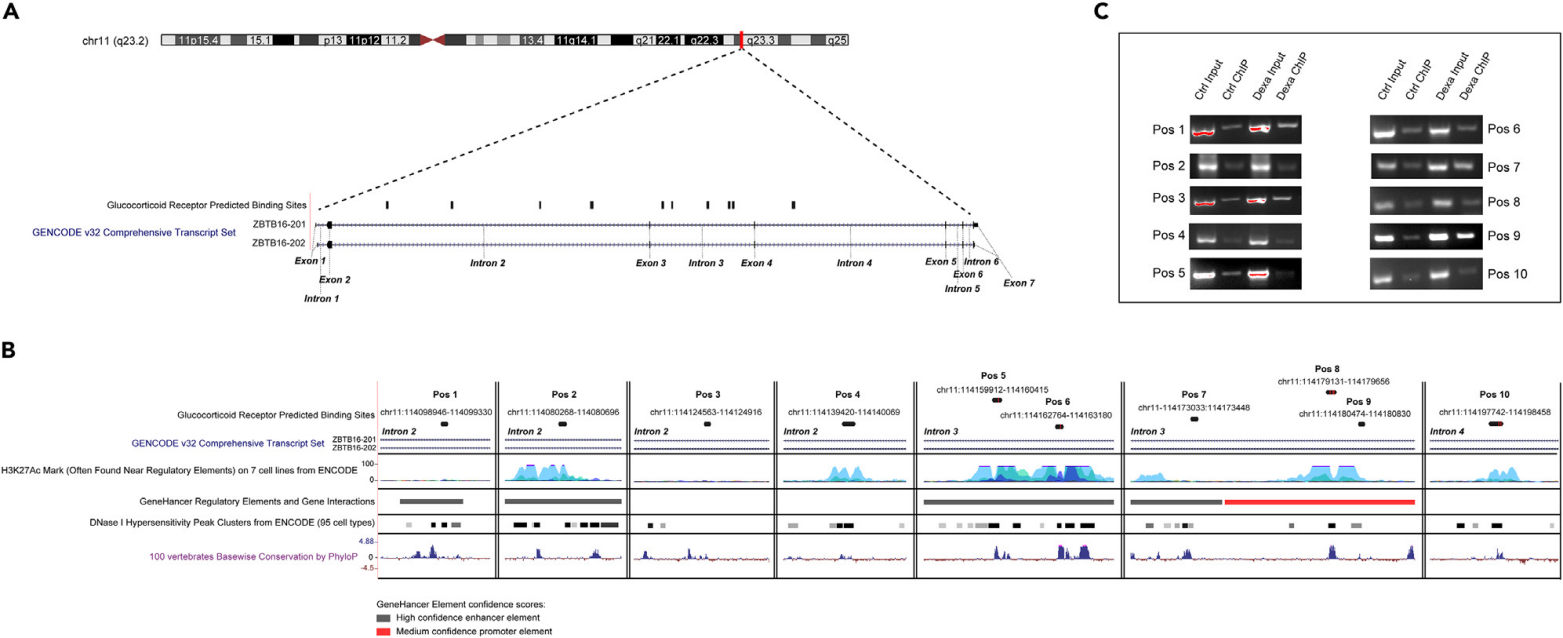
Glucocorticoid responsive elements (GREs) are located in intronic and conserved regions of *ZBTB16* in human β-cells (A) Distribution of predicted GREs across *ZBTB16* gene on the human islets. (B) Predicted GRE overlaps (and their genomic coordinates) visualized with regulatory/conservation tracks from UCSC Genome Browser; tracks represent from top to bottom: ZBTB16 transcript localization, ENCODE regulatory elements, GeneHancer regulatory elements, Open chromatin positions (DNase I peak clusters), Phylop basewise evolutionary/conservation scores. Red lines on the peak boxes (positions 5, 6, 8, and 10) correspond to GR binding sequence (GBS) occurrences. (C) Glucocorticoid Receptor ChIP followed by PCR for the predicted GRE coordinates. Position numbers correspond to the positions of (A) from left to right (see also Figure S1). Ctrl; Control (DMSO), Dexa; Dexamethasone (100 nM).

Next, to uncover the genomic positions that could potentially lead to *ZBTB16* expression induction in the human β-cells, we listed the GREs that were associated with *ZBTB16* in open chromatin regions that were common between human islets and EndoC-βH1 cells. Although the GRE search spanned a 150 kb window around *ZBTB16* TSS, only 10 intronic positions satisfied the criteria (Figure 4A). When the 10 positions were visualized in the University of California, Santa Cruz (UCSC) Genome Browser together with open chromatin, regulatory, and conservation tracks on the same coordinates, 9 of 10 positions overlapped with comprehensive open chromatin and regulatory positions, and 5 of 10 positions co-localized with highly conserved regions (Positions: 5–8,10) (Figure 4B). Moreover, GBS occurrences were detected on 4 GREs (Positions: 5, 6, 8, and 10; positions 8 and 10 contain more than one GBS), all of which are located in highly conserved regions. Taken together, we hypothesized that the induction of *ZBTB16* expression is possibly related to the differential binding of GR to one or several of the *ZBTB16* identified GREs. To prove our hypothesis, we performed ChIP followed by PCR in EndoC-βH1 cells pre-treated in the absence or presence of 100 nM dexamethasone for 24h. GR binding was validated in all the 10 *ZBTB16* GREs (Figure 4C) and we observed a consistent active binding in the treated samples compared to the non-treated samples, although with some degree of variability (Figure S1). The presence of multiple GREs within *ZBTB16* suggests a highly dynamic transcriptional regulation of this gene resulting in its hyperactivation upon glucocorticoid treatment.

### Dexamethasone induces *ZBTB16* expression in a dose- and time-dependent manner and impairs insulin secretion in EndoC-βH1 cells

**Figure 5:**
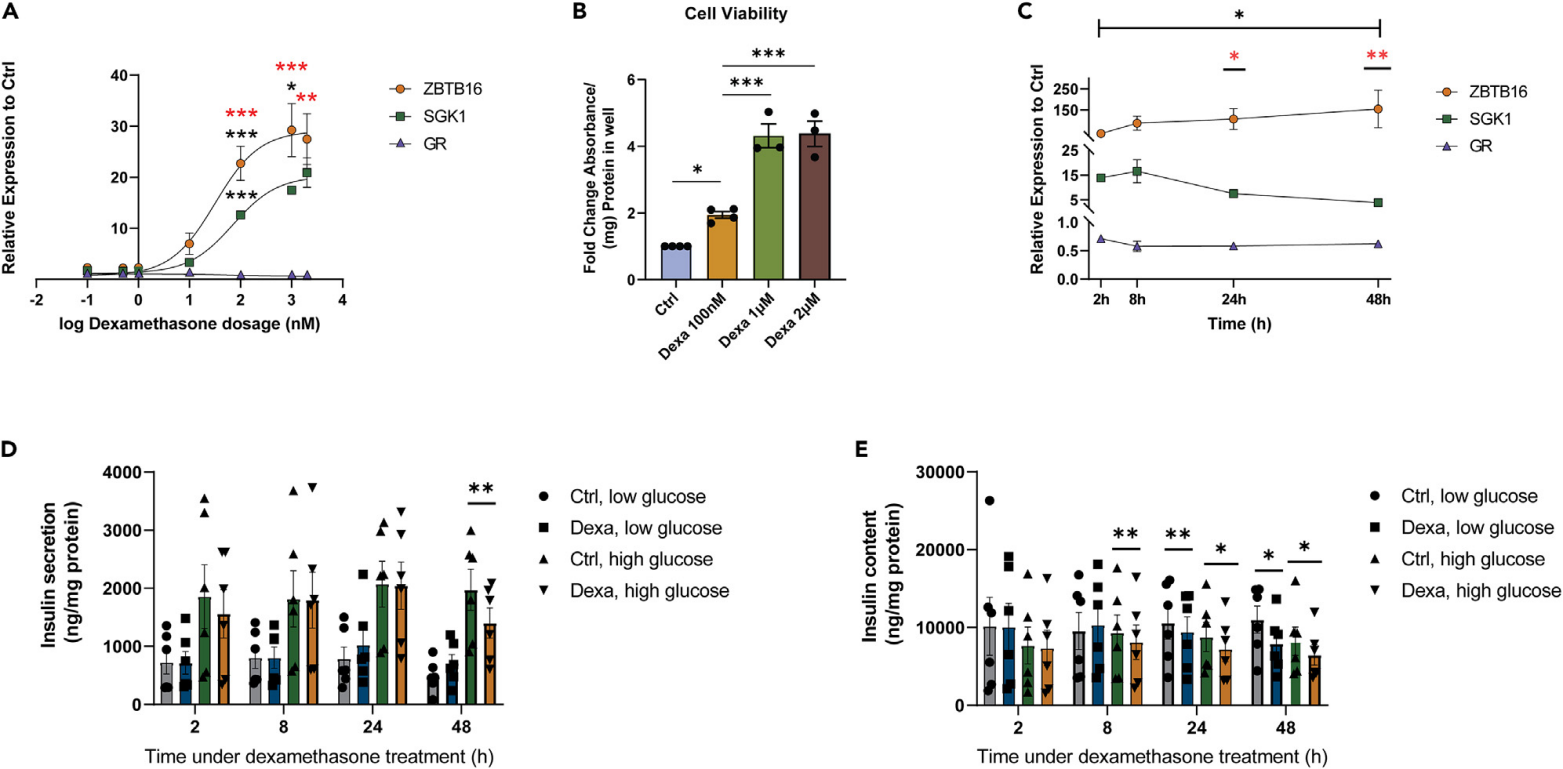
Dexamethasone induces *ZBTB16* expression in a dose- and time-dependent manner and impairs insulin secretion in EndoC-βH1 cells (A) Dose-response curve of increasing concentration of dexamethasone treatment (0.1, 0.5, 1, 10, 100, 1000, and 2000 nM) for 24h in the expression levels of *ZBTB16*, *SGK1,* and *GR* (n = 4 biological replicates). Black stars indicate gene-specific expression differences between successive doses (e.g., 1 vs. 10, 10 vs. 100, 100 vs. 1000, and 1000 vs. 2000 nM). Red stars illustrate expression differences between *ZBTB16* and *SGK1*. *ZBTB16* and *SGK1* expression levels are significantly different from that of *GR* in all treatment doses (significance symbols omitted for clarity). (B) Cell viability (MTS) assay relative to the control after treatment with increasing dexamethasone concentrations (n = 4 biological replicates for 100 nM, 3 biological replicates for 1 and 2 μΜ) for 48h. (C) Gene expression levels of *ZBTB16*, *SGK1,* and *GR* upon treatment with 100 nM dexamethasone at different time points (2, 8, 24, and 48h) (n = 4 biological replicates). The black star indicates gene-specific expression difference at each time point compared to 2h post-treatment. Red stars illustrate expression differences between *ZBTB16* and *SGK1*. *ZBTB16* and *SGK1* expression levels are significantly different from that of *GR* in all time points (significance symbols omitted for clarity). (D) Insulin secretion of EndoC-βH1 cells upon treatment with 100 nM dexamethasone at different time points (n = 6 biological replicates). All comparisons between low and high glucose treatments were significantly different regardless of treatment but the symbols for significance were omitted for clarity. (E) Insulin content of EndoC-βH1 cells upon treatment with 100 nM dexamethasone at different time points (n = 6 biological replicates). Forevery grouped comparison repeated measures ANOVA (1-, 2-, or 3-way) was performed. Post-hoc pairwise comparisons included Tukey multiple comparisons for cell viability, dose- and time-dependent dexamethasone treatment experiments and paired t-tests for insulin secretion and insulin content experiments. Low glucose; 1 mM, high glucose; 20 mM, Ctrl; Control (DMSO), Dexa; Dexamethasone. Data are presented as mean ± SEM; ∗p < 0.05, ∗∗p < 0.01, ∗∗∗p < 0.001.

To further investigate the conditions under which *ZBTB16* transcription is induced in the β-cells, we measured the expression levels of *ZBTB16* after treatment with different doses of dexamethasone (0.1–2000 nM) for 24h in EndoC-βH1 cells (Figure 5A). The *ZBTB16* expression at the different concentrations of dexamethasone was compared with those of genes that are known to be involved in the glucocorticoid-GR signaling pathway in β-cells, such as *SGK1* (activated) and *GR* (repressed).^16^ As shown in Figure 5A, there were significant differences in the expression of the genes that depended on glucocorticoid dose (two-way ANOVA: *gene expression∗dose* p < 0.001). The pairwise comparisons showed an increasing expression of *ZBTB16* in response to dexamethasone concentration in the range of 10–1000 nM, which is higher than that of *SGK1*. Since 100 nM appeared to be the lowest dexamethasone concentration in which we observed significant target gene induction, we used this concentration as our working concentration in subsequent functional experiments. At the same time, treatment with increasing dexamethasone concentrations also led to a significantly elevated proliferation rate (Figure 5B).

We next measured the expression of the same genes in EndoC-βH1 cells after incubation with or without 100 nM dexamethasone for an increasing time period (2h, 8h, 24h, and 48h; Figure 5C). The results demonstrated significant differences in the expression of genes that depend on time under treatment (two-way ANOVA: *gene expression∗time* p < 0.05, Figure 5C). Pairwise comparisons reveal that *ZBTB16* expression is significantly induced 48h post-treatment (compared to 2h post-treatment) and it is significantly more expressed than *SGK1* 24h and 48h post-treatment.

We also asked whether the treatment with 100 nM dexamethasone has a time-dependent effect on glucose-stimulated insulin secretion. For that reason, we performed insulin secretion assay in the presence of 1- and 20-mM glucose in EndoC-βH1 cells after incubation with 100 nM dexamethasone at different time points (2h, 8h, 24h, and 48h). Analysis showed that insulin secretion was significantly different depending on the glucose concentration and incubation time (3-way ANOVA: *insulin secretion∗glucose concentration∗time* p < 0.001, Figure 5D). Pairwise comparisons showed that glucose-stimulated insulin secretion is significantly reduced after a 48h dexamethasone treatment period. Dexamethasone caused a significant reduction of the cellular insulin content across time under treatment (3-way ANOVA: *insulin content∗time* p < 0.05), which is apparent and consistent 8h post-treatment (Figure 5E). This can partly explain the reduced insulin secretion that was observed.

### Induction of *ZBTB16* expression can be protective for the β-cell

**Figure 6:**
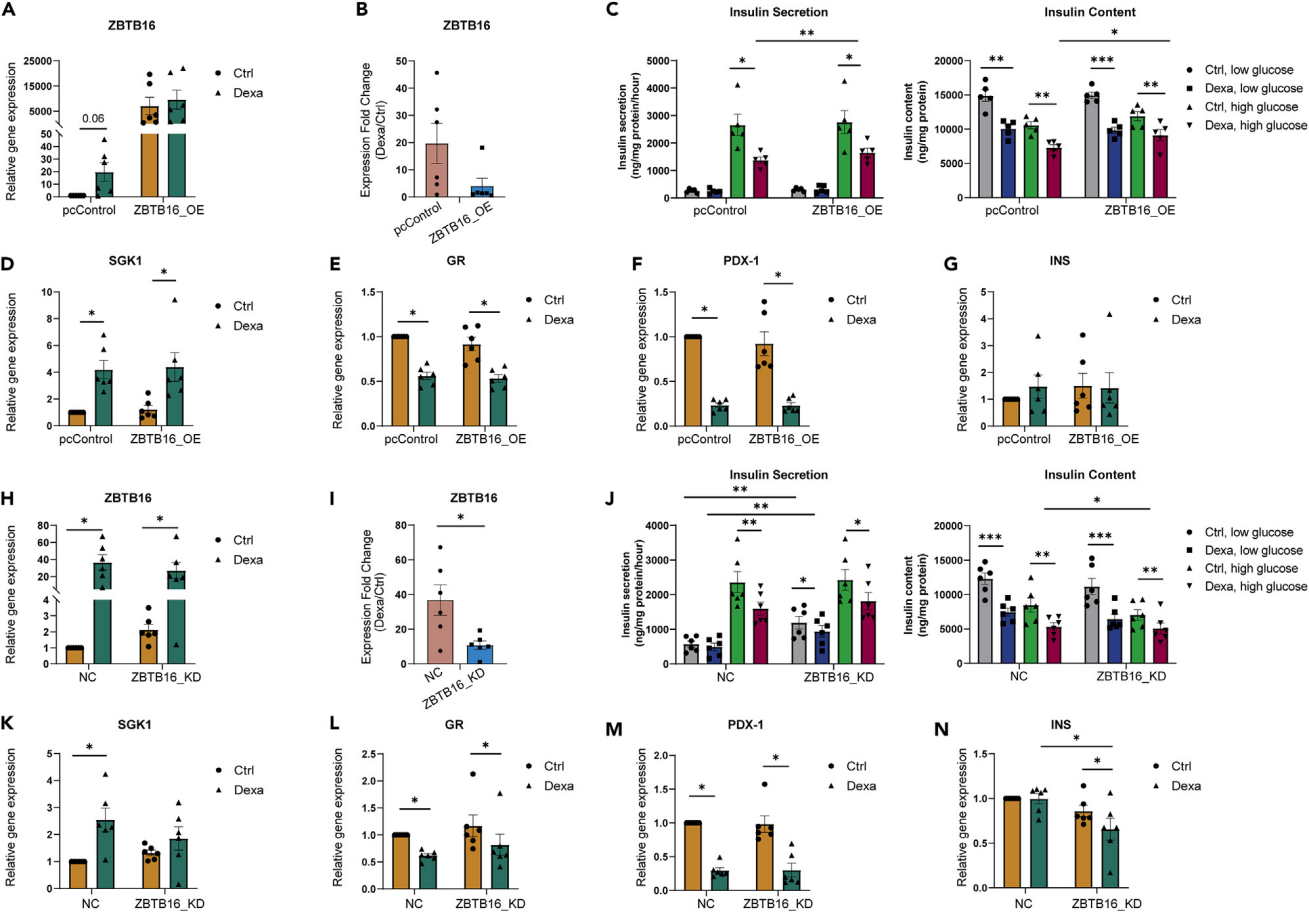
*ZBTB16* overexpression and induction suppression affect gene expression and insulin secretion in EndoC-βΗ1 cells (A) Relative expression of *ZBTB16* (compared to Ctrl) after *ZBTB16* overexpression as measured by qPCR assay. (B) Expression induction of *ZBTB16* (fold-change relative expression; Dexa/Ctrl expression) in control and dexamethasone-treated samples after *ZBTB16* overexpression. (C) Insulin secretion (left) and insulin content (right) after *ZBTB16* overexpression. (D) Relative expression of *SGK1*, (E) *GR*, (F) *PDX-1*, (G) *INS* (compared to Ctrl) after *ZBTB16* overexpression as measured by qPCR assay. (H) Relative expression of *ZBTB16* (compared to NC) after *ZBTB16* induction suppression as measured by qPCR assay. (I) Expression induction of *ZBTB16* (fold-change relative expression; Dexa/Ctrl expression) in NC and dexamethasone-treated samples after *ZBTB16* induction suppression. (J) Insulin secretion (left) and insulin content (right) after *ZBTB16* induction suppression. (K) Relative expression of *SGK1*, (L) *GR*, (M) *PDX-1*, (N) *INS* (compared to NC) after *ZBTB16* induction suppression as measured by qPCR assay. All experiments were conducted on EndoC-βH1 cells (n = 5–6 biological replicates). Comparisons between qPCR results were performed using the Wilcoxon matched pairs signed rank test. Insulin secretion and content grouped comparisons were performed with repeated measures two-way ANOVA followed by pairwise comparisons with paired Student’s *t* test. Low glucose; 1 mM, high glucose; 20 mM, Ctrl; Control (DMSO), Dexa; Dexamethasone (100 nM), pcControl; Control plasmid pcDNA3.1, ZBTB16_OE; *ZBTB16* overexpression, NC; Negative Control, ZBTB16_KD; *ZBTB16* knockdown. Data are presented as mean ± SEM; ∗p < 0.05, ∗∗p < 0.01, ∗∗∗p < 0.001.

We were next interested in whethter increased levels of *ZBTB16* are partly responsible for the deleterious negative effects of glucocorticoid treatment on insulin secretion. We, therefore overexpressed *ZBTB16* in EndoC-βH1 cells, achieving a high level of induction (Figures 6A-B). Cells were then incubated in the absence or presence of 100 nM dexamethasone for 48h before insulin secretion assay is performed. Overexpression of *ZBTB16* affected glucose-stimulated insulin secretion independently of the dexamethasone treatment (3-way ANOVA: *ZBTB16 overexpression ∗ dexamethasone treatment ∗ glucose concentration* p = 0.13, *ZBTB16 overexpression* p = 0.006) by slightly rescuing the dexamethasone-induced impairment of insulin secretion (Figure 6C-left). This effect was accompanied by increasing cellular insulin content (Figure 6C-right).

We investigated whether the induction of *ZBTB16* could be involved in gene regulatory programs mediated by the glucocorticoid-GR regulatory axis in β-cells, by determining the gene expression of the glucocorticoid gene targets *SGK1*, *GR*, *PDX-*1, as well as the insulin gene (*INS*). *ZBTB16* induction did not alter the expression of any of those genes (Figures 6D–G), suggesting that *ZBTB16* is not mediating the glucocorticoid-induced expression changes of *SGK1* or *PDX-1*.

Next, we attempted to suppress *ZBTB16* induction after dexamethasone treatment and measure glucose-stimulated insulin secretion and expression of the glucocorticoid gene targets. Knockdown of *ZBTB16* under this condition led to a partial *ZBTB16* suppression of induction by ≈ 30% (Figures 6H-I). We found that the partial reduction in *ZBTB16* expression influenced insulin secretion independently of the dexamethasone treatment and glucose concentration (3-way ANOVA: *ZBTB16 suppression ∗ dexamethasone treatment ∗ glucose concentration* p = 0.2, *ZBTB16 suppression* p = 0.002) and it led to increased insulin secretion under low glucose treatment both in the control and the treated samples (Figure 6J-left). Insulin content was found to be reduced in the *ZBTB16*-suppressed samples compared to the control (Figure 6J-right). Partial suppression also caused changes in the glucocorticoid-mediated gene expression patterns, as *SGK1* induction was inhibited (Figure 6K). However, expression of *GR* and *PDX-1* were unaltered under the same condition (Figure 6L-M). Insulin transcription was reduced in the treated cells with partially suppressed *ZBTB16* (Figure 6N), which is in line with the observed reduction of insulin content.

**Figure 7:**
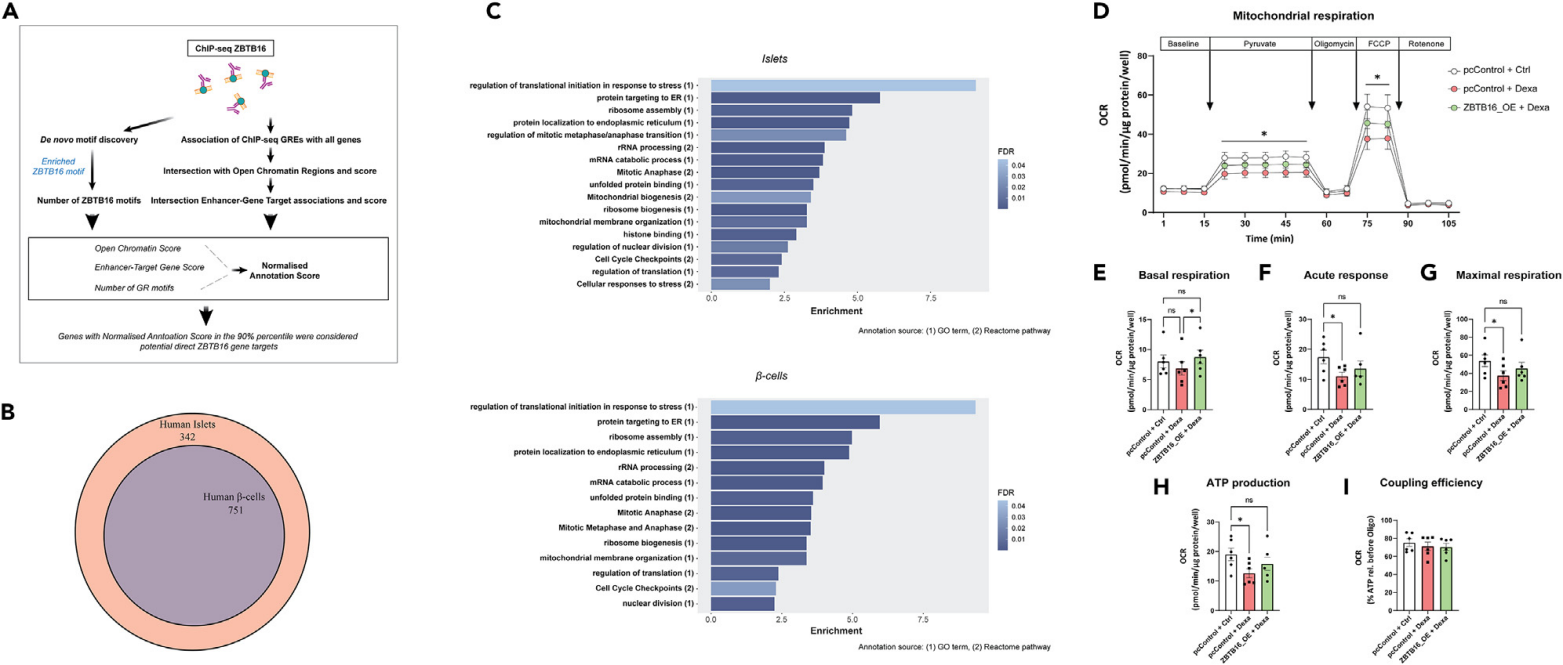
ZBTB16 predicted direct gene targets are involved in several regulatory pathways, including mitochondrial function (A) Schematic summary of the bioinformatics workflow to identify ZBTB16 predicted direct gene targets in human pancreatic islets. (B) Subset of potential islet ZBTB16 targets expressed in human pancreatic β-cells. (C) Bar chart showing selected enriched terms/pathways derived from the functional annotation of the ZBTB16 predicted direct gene targets in human islets (*Up*) and human β-cells (*Down*). (D) Mitochondrial oxygen consumption measurements in EndoC-βH1 cells (n = 6 biological replicates) corresponding to cellular respiration levels comparing control cells (pcControl + Ctrl), control cells under dexamethasone treatment (pcControl + Dexa) and cells with overexpression of *ZBTB16* under dexamethasone treatment (ZBTB16_OE + Dexa), (E) Quantification of baseline OCR levels, (F) Acute response measured as increase in OCR upon pyruvate injection, (G) Maximal respiration measured after addition of an uncoupler of the inner mitochondrial membrane (FCCP), (H) ATP production assessed as decrease in OCR after oligomycin injection, which inhibits ATP-synthase and (I) Coupling efficiency calculated as ATP production in relation to combined baseline and pyruvate response. Comparisons between conditions in each measurement were performed with one-way ANOVA followed by pairwise comparisons with Tukey’s multiple comparison test. pcControl; Control plasmid pcDNA3.1, ZBTB16_OE; ZBTB16 overexpression, Ctrl; Control (DMSO), Dexa; Dexamethasone (100 nM), FCCP; carbonyl cyanide p-trifluoro-methoxyphenyl hydrazone. Data are presented as mean ± SEM; ∗p < 0.05.

Furthermore, to evaluate the cellular function of ZBTB16 we used our bioinformatics pipeline to identify potential direct gene targets of ZBTB16 in human islets (Figure 7A and STAR Methods). The analysis detected 1093 predicted gene targets (Table S4 - tab A). By analyzing the transcriptome of human islet β-cells derived from a published single-cell study (GEO: GSE153855) we found that 751 of these targets are also expressed in the human β-cells (Figure 7B and Table S4 - tab B). Functional annotation of the ZBTB16 target genes expressed in either human islets or β-cells revealed similar enriched terms/pathways related to rRNA and mRNA processing, ER protein targeting, cell cycle regulation, and mitochondrial membrane organization among others (Figure 7C, full lists in Table S5). Thus, we next sought to get a deeper understanding of the mechanism of action of ZBTB16 by investigating cell proliferation and mitochondrial function in EndoC-βH1 cells under dexamethasone treatment after ZBTB16 manipulation. As mentioned before, the proliferation rate after dexamethasone treatment is considerably increased (Figure 5B) and ZBTB16 induction or suppression did not seem to have any additional effects (Figure S3).

Regarding mitochondrial function, we performed mitochondrial oxygen consumption rate (OCR) measurements in EndoC-βH1 cells, which were assessed as whole cell OCR after overexpressing *ZBTB16* and treating with dexamethasone. The overall effect of dexamethasone was a clear suppression of mitochondrial respiration, indicating impaired mitochondrial function, as outlined by a general lowering of OCR during pyruvate and carbonyl cyanide p-trifluoro-methoxyphenyl hydrazone (FCCP) measurements across the entire recording trace (Figure 7D). This suppression was no longer significant in EndoC-βH1 cells overexpressing *ZBTB16* under dexamethasone treatment, indicating that overexpression of *ZBTB16* rescues mitochondrial function. Basal respiration was found to be higher in cells overexpressing *ZBTB16* under dexamethasone treatment than those in control cells exposed or not in dexamethasone (Figure 7E). Acute response measured as increase in OCR upon pyruvate injection, ATP production assessed as decrease in OCR after oligomycin injection that inhibits ATP-synthase, and maximal respiration as measured after the addition of an uncoupler of the inner mitochondrial membrane (FCCP), all showed the same trend where dexamethasone treatment consistently led to significantly reduced magnitude of OCR responses to these injections (Figures 7F–H). Overexpression of *ZBTB16*, however, did not show the same degree of reduction, once again showing that *ZBTB16* can rescue these aspects of mitochondrial function. Coupling efficiency was not altered by dexamethasone treatment or by overexpressing *ZBTB16* (Figure 7I).

## Discussion

Multiple studies have highlighted the contribution of the pharmacological use of glucocorticoids as a mediator of T2D development, including impaired insulin secretion. However, a comprehensive understanding on how glucocorticoids affect β-cell function has not been fully reached. In this study, we performed RNA-seq analysis that allowed us to compare transcriptomic changes after glucocorticoid treatment in human pancreatic islets and the human insulin-secreting cell line EndoC-βH1. Integration of our transcriptomic results with ChIP-seq GR binding, open chromatin state, and enhancer data from publicly available resources allowed us to study GR binding properties in both human islets and EndoC-βH1 cells and identify *ZBTB16* as a highly confident direct gene target of GR. Finally, we functionally verified that *ZBTB16* is 1) strongly induced in EndoC-βH1 cells and 2) most likely has a protective role by increasing insulin secretion, limiting the otherwise deleterious reduction of glucose-stimulated insulin secretion by glucocorticoids.

Dexamethasone was selected as the synthetic glucocorticoid to perform all *in vitro* experiments in this study. This was because several studies that were focused on the glucocorticoid effect in human islets or human β-cell lines^16^^,^^23^^,^^33^ or the glucocorticoid genomic response in other human cell lines^38^^,^^39^^,^^40^ also utilized dexamethasone and, in that way, we would be able to directly compare, evaluate, and discuss the conclusions of those studies more accurately. Interestingly, approximately 40% of the DE genes in human islets after dexamethasone treatment were differentially expressed in the human β-cell line EndoC-βH1. Functional annotation analysis revealed these genes to be involved in pathways crucial for β-cell function and development. Many of these pathways are already known to be implicated in glucocorticoid-mediated impaired insulin secretion in rodent islets or cell lines. These include glucose metabolism,^41^ increased potassium channel activity,^24^ calcium transport and insulin secretion,^12^^,^^13^^,^^41^ β-cell proliferation,^42^^,^^43^ and insulin processing.^44^ Other pathways such as pancreas development, differentiation and lipid metabolic process, and localization have not been previously studied in a β-cell context to the best of our knowledge. Hence, the effect on gene expression between human islets and EndoC-βH1 cells has many similarities. Moreover, among the distinctly DE genes between islets and EndoC-βH1 cells several of them are involved in identical pathways related to glucose metabolism, insulin secretion, and translational modifications, while other genes in unrelated pathways. Human islet-specific glucocorticoid-induced changes, such as genes related to insulin response, may be associated with the existence of other pancreatic cell types in the islets, such as the α-cells, as glucagon secretion has been found to be reduced in mouse islets after acute dexamethasone treatment.^45^

The molecular mechanisms regulating glucocorticoid-induced changes in gene expression are complex and can differ between cell types.^46^^,^^47^ Here, by using ChIP-seq data we showed that the majority of GRE sites are located further than 3 kb away from the TSS of the associated genes, confirming that GR is not characterized by the typical promoter-proximal binding pattern.^39^^,^^48^ Furthermore, a significantly higher number of GREs were detected closer to upregulated or downregulated DE genes than non-DE genes, supporting a role of GR as both an activator and repressor of gene expression.^49^^,^^50^ It was also apparent from our data that GR binding was more strongly associated with the induced rather than the repressed DE genes, which is consistent with results from GR global recruitment experiments in other human cell lines.^39^^,^^51^ Although this may imply that the mechanism underlying GR repression depends on long-range interactions between a smaller number of more distal GREs and the TSS of target genes,^39^ other studies suggest that the lower GRE abundance near downregulated DE targets may be due to the GR binding to distinct negative GREs (nGREs) with low-affinity,^52^ which are untraceable by ChIP-seq peak calling methods because of their weak ChIP-seq signal.^53^

To get a deeper understanding of the Glucocorticoid Receptor Binding Sequence (GBS) within the GREs near the glucocorticoid-regulated DE genes in islets and EndoC-βH1, we performed a *de novo* motif discovery. Only a proportion (≈77%) of GREs seems to encompass the standard 15-bp GBS,^54^ consistent with previous reports indicating a similar proportion (60–80%) on different human cell lines.^8^^,^^39^^,^^55^ This could explain why a small fraction of classical GBSs in the genome is actually occupied by GR.^48^ Our data also demonstrates the important role of additional TFs in GR gene regulation. The vast majority of the GREs in our data (95%) were found to be composite response elements containing at least one alternative motif beside the GBS, indicating that a big part of GR regulation depends on the binding of auxiliary TFs, other than GR, on the GREs.^56^ GREs were enriched for motifs similar to those of the AP-1, ETS/TEAD, and FOX gene families. In another study, computational analysis using ChIP-exo data revealed that FOX factors seem to co-occupy the GREs along with GR, possibly to maintain the open chromatin conformation and allow GR to bind to the genome.^40^ The function of AP-1, on the other hand, appears to be limited to providing access to chromatin without directly interacting with the GR on the GREs.^40^ In the same study, it was also demonstrated that members of the ETS/TEAD protein families directly interacted with GR and tethered it to the DNA.^40^ The regulatory attributes of these elements explain why their motif binding sequences are enriched not only in the GREs of our ChIP-seq sets but in other GRE ChIP-seq studies too.^40^^,^^55^^,^^57^^,^^58^

With the intention of identifying potential direct GR targets among the list of DE genes, distinct types of publicly available data were integrated into a custom bioinformatics pipeline, which allowed the ranking of the genes according to how strong the evidence that suggests direct targeting by GR was. The top-ranked DE genes included well-characterized GR targets such as *FKBP5*^59^ and *VIPR1*,^33^^,^^60^ as well as other known islet/β-cell-specific genes such as *PDX-1* and *NKX6-1*,^16^ the latter indicating that these results are trustworthy. The top-ranked DE gene in both human islet and EndoC-βH1 samples was *ZBTB16*, which was recently demonstrated to be induced by dexamethasone in pancreatic islets.^33^ The *ZBTB16* gene has also demonstrated the highest degree of methylation alterations after dexamethasone treatment in insulin-secreting cells.^23^ Previous studies have also linked *ZBTB16* with systemic glucose homeostasis as a negative regulator of adipogenesis^61^ and insulin sensitivity in skeletal muscle and liver.^62^^,^^63^

When we investigated the GR binding to *ZBTB16* in order to understand the molecular regulation of its function, we found contrary to common belief (regulatory elements reside mostly upstream or downstream of their target genes) that the 10 GREs associated with *ZBTB16* were located exclusively on intronic sites of the gene. Nevertheless, intronic GR binding has been recorded before in a relatively high fraction (25–30%) of the total identified GREs,^39^^,^^58^ with luciferase reporter assay results suggesting a potential functional role of the GBSs of intronic GREs.^64^^,^^65^ Moreover, while the majority of regions reside in genomic regulatory regions, only 4 GREs in intron 3 and 1 GRE in intron 4 were overlapping with highly conserved regions and contain at least one GBS. The central role of these positions in the glucocorticoid-mediated regulation of *ZBTB16* was also supported by the significant induction of open chromatin signal in these regions in human islets, which were treated with both high and low doses of dexamethasone.^33^ ChIP followed by PCR in EndoC-βH1 cells revealed a consistent active binding of GR in nine out of the ten predicted GREs, although with some degree of variation between the replicates. The fact that fluctuating levels of islet-specific accessible chromatin signal were also observed in different replicates in control and dexamethasone-treated human islets,^33^ indicates a rather dynamic GR regulatory control on *ZBTB16* that may depend on GR interaction with TFs that modulate chromatin accessibility. The involvement of auxiliary TFs in the glucocorticoid-GR signaling pathway could also explain the existence of more GR-bound regions without a GBS (six out of ten) than regions with a GBS on the introns of *ZBTB16*. As at least five out of the ten predicted GR-bound regions on *ZBTB16* reside in non-conserved genomic regions (Figure 4, positions: 1–4, 9), it is probable that these regions constitute cell-type specific regulatory elements.

Finally, we disclosed the expression induction of *ZBTB16*, which characterizes the glucocorticoid effect, on the β-cell function by manipulation of *ZBTB16* expression in EndoC-βH1 cells and prediction of direct islet-specific *ZBTB16* gene targets with bioinformatics methods. Gene expression assays on EndoC-βH1 showed a significant induction of *ZBTB16* expression after dexamethasone treatment that was dependent on the dose and the time under treatment. After incubating EndoC-βH1 cells with dexamethasone, impairment of insulin secretion under high glucose conditions was observed after 48h, which was preceded by decreased cellular insulin content 8h post-treatment. The reduction of insulin content together with the observed increased proliferation rate may suggest that the cells are transitioning to a lower maturity state as has been shown before in c-Myc-expressing INS1 cells.^66^ This is supported by the functional annotation of DE genes after dexamethasone treatment where *β-cell pancreatic cell differentiation* is significantly enriched (Figure 1C). Moreover, taking into account that *ER stress* is the top enriched molecular pathway of DE genes after dexamethasone treatment in EndoC cells (Figure 1C), we hypothesize that reduced insulin content may be a compensatory mechanism of EndoC-βH1 cells against elevated ER stress levels.^67^

Overexpression of *ZBTB16*, though, could moderately rescue cellular insulin content and secretion. Moreover, after partially suppressing the dexamethasone-mediated *ZBTB16* expression induction, we noticed increased insulin secretion under low glucose conditions, resembling the diabetic phenotype.^68^ In the same samples, *INS* expression was reduced and *SGK1* gene induction, which has been associated with insulin release and type 2 diabetes,^24^^,^^69^ was inhibited. Everything considered, increased expression of *ZBTB16* through induction by glucocorticoids may have a protective role hampering the otherwise deleterious effects of glucocorticoids in the β-cell.

Taking one step further, our bioinformatics pipeline also revealed potential direct targets of *ZBTB16* in the human islets. Integration of β-cell transcriptome data revealed that 68% of these targets are also expressed in the human pancreatic β-cell. Despite this divergence, pathway analysis uncovered a similar group of significantly enriched terms related to mitochondrial membrane/biogenesis, regulation of translation, and cell cycle/division in both human islet and β-cell targets.

*ZBTB16* overexpression has been previously associated with mitochondrial number and function in brown adipocytes.^70^ The fact that in fasting conditions *ZBTB16* expression is suppressed in β-cells in a similar fashion to the energy-storing white adipose tissue, and is induced in tissues with high-energy requirements such as the brown adipose tissue and skeletal muscle,^62^ could imply its implication in stress responses that aim to cover the energy demands of the cell. In EndoC-βH1 cells, while we found dexamethasone treatment negatively affected mitochondrial function, confirming findings from previous studies in insulin-secreting and other cell lines,^71^^,^^72^ we also found that overexpression of ZBTB16 could completely compensate for these negative effects and restore mitochondrial function to normal levels. As insulin secretion is well known to be coupled to mitochondrial function, these results go in line with the insulin secretion measurements showing that ZBTB16 can restore insulin secretion, reinforcing its protective role in the β-cell. The implication of ZBTB16 in a protective β-cell mechanism via improved mitochondrial function has been shown before in rats undergoing far-infrared radiation.^73^ *ZBTB16* predicted targets annotated as relevant to mitochondrial membrane and biosynthesis include *MED1*^74^ and *TMEM11*.^75^

Regarding cell cycle control, *ZBTB16* has been reported as both negative and positive regulator of cell cycle control in different cell types.^76^^,^^77^^,^^78^^,^^79^^,^^80^ In our hands, ZBTB16 overexpression or suppression in EndoC-βH did not alter cell viability/proliferation in control conditions and did not have any additive effects on the significantly increased cell proliferation rate upon dexamethasone treatment. In that context, several genes including *SPDL1*,^81^ *PDS5A,*^82^ and *SLC12A2*^83^ were identified as targets that can modulate cell division. It is also worth mentioning that miR-375, a miRNA that is important for β-cell development, proliferation, and secretion^84^ was also among the *ZBTB16* targets. Among the targets of ZBTB16 there were several genes implicated in the post-transcriptional regulatory mechanism. For instance, *ATF4*^85^ and *MAPK14* (alias: *p38*)^86^ both mediate ER function upon cellular stress and *AKT2* phosphorylates targets involved in protein synthesis.^87^

Taken together, glucocorticoids trigger distinct transcriptome changes in human islets and EndoC-βH1 cells, although with significant similarities. GR binding patterns display considerable similarities between the two and seem to be largely dependent on the interplay with other transcription factors. *ZBTB16* is the most highly confident direct GR genetarget in both human islets and EndoC-βH1 cells and its substantial glucocorticoid induction may be able to alleviate the cellular stress by improving mitochondrial function as a part of a compensatory mechanism against the β-cell dysfunction triggered by other glucocorticoid targets. Altogether, this study provides a better insight on the mode of function of glucocorticoids in the pancreatic islets and β-cells, which should be valuable when developing pharmacological treatment strategies against glucocorticoid-induced diabetes in the future.

### Limitations of the study

A caveat of the current study is the low number of human islet samples that were used for RNA-sequencing after GC treatment (n = 4 human donors) due to the low availability of such samples. This was the reason that further functional investigation on the *ZBTB16* gene was performed only in EndoC-βH1 cells in order to ensure satisfactory number of repeats (n = 4–6 biological replicates/assay). Moreover, we attempted to define GR binding properties in human islets and β-cells by using publicly available ChIP-GR binding sites from other tissues and cell types. We addressed this issue by retaining the positions, which overlapped with islet and β-cell open (accessible) chromatin regions. Despite being able to validate these positions on the *ZBTB16* gene in EndoC-βH1 cells, there may be additional cell-type specific GR binding positions that may be revealed only with more targeted experimental approaches such as GR ChIP-seq in islets and β-cells. Finally, we demonstrated a protective effect of *ZBTB16* against the deleterious effects of dexamethasone in EndoC-βH1 cells. However, further investigation is necessary in human islets and *in vivo* animal models in order to more robustly assess the compensatory beneficial effects of *ZBTB16* after glucocorticoid treatment.

## STAR★Methods

### Key resources table

**Table:** 

REAGENT or RESOURCE	SOURCE	IDENTIFIER
**Antibodies**		

ZBTB16 (PLZF) (D8G3G) Rabbit mAb	Cell Signaling Technology	#39784
Anti-rabbit IgG, HRP-linked	Cell Signaling Technology	#7074
Glucocorticoid Receptor (D6H2L) XP® Rabbit mAb	Cell Signaling Technology	#12041

**Biological samples**		

Human pancreatic islets from healthy donors and donors with T2D	Nordic Network for Clinical Islet Transplantation, Human Tissue Laboratory, EXODIAB/LUDC (http://www.nordicislets.org).	N/A

**Chemicals, peptides, and recombinant proteins**		

Dexamethasone	Sigma-Aldrich	#D1756
ZBTB16 TaqMan® gene expression assay	Thermo Fisher	Hs00232313_m1
GR TaqMan® gene expression assay	Thermo Fisher	Hs00353740_m1
SGK1 TaqMan® gene expression assay	Thermo Fisher	Hs00353740_m1
PDX-1 TaqMan® gene expression assay	Thermo Fisher	Hs00236830_m1
INS TaqMan® gene expression assay	Thermo Fisher	Hs02741908_m1
4–15% Mini-PROTEAN® TGX Stain-Free™ Protein Gels	Bio-Rad	#4568084
Clarity Western ECL Substrate	Bio-Rad	#1705061
GelRed® Nucleic Acid Stain	Sigma-Aldrich	#SCT123
Lipofectamine LTX	Thermo Fisher	#15338-100
Lipofectamine™ RNAiMAX Transfection Reagent	Thermo Fisher	#13778075
3-Isobutyl-1-methylxanthine (IBMX)	Sigma-Aldrich	#I5879
Dimethyl sulfoxide (DMSO)	Sigma-Aldrich	#34869
Sodium pyruvate	Sigma-Aldrich	#P2256
Oligomycin A	Sigma-Aldrich	#75351
Carbonyl cyanide 4-(trifluoromethoxy)phenylhydrazone (FCCP)	Sigma-Aldrich	#C2920
Rotenone	Sigma-Aldrich	#R8875

**Critical commercial assays**		

miRNeasy isolation kit	Qiagen	#217084
TruSeq Stranded Total RNA Library Prep with Ribo-Zero kit	Illumina	#RS-122-2201
High-Capacity cDNA Reverse Transcription Kit	Thermo Fisher	#4368814
Nextseq 500/550 High Output kit v2	Illumina	#FC-404-2002
Pierce™ BCA Protein Assay Kit	Thermo Fisher	#23225
ChIP-IT® Express Enzymatic kit	Active Motif	#53009
Insulin ELISA (Human)	Mercodia	#10-1113-01
CellTiter 96® AQueous One Solution Cell Proliferation Assay	Promega	#G3582

**Deposited data**		

Raw and processed RNA sequencing data from human islet and EndoC-βH1 cells upon dexamethasone treatment	This paper	GEO: GSE225901

**Experimental models: Cell lines**		

EndoC-βΗ1	Ravassard et al.^88^	RRID:CVCL_L909

**Oligonucleotides**		

Primers for GR binding sites on ZBTB16 gene, see Table S7	This paper	N/A
ZBTB16 Silencer Select Pre-Designed siRNA	Thermo Fisher	n341241; #4390771
Silencer Select Negative Control No. 2 siRNA	Thermo Fisher	#AM4613

**Recombinant DNA**		

ZBTB16_OHu04229C_pcDNA3.1(+)-C-HA	GeneScript	#SC1200

**Software and algorithms**		

Adobe Illustrator (23.0.1)	Adobe	https://www.adobe.com/products/illustrator.html, RRID:SCR_010279
FastQC (0.11.8)	Andrews et al.^89^	RRID:SCR_014583
Salmon (0.14.0)	Patro at al.^90^	RRID:SCR_017036
Deseq2 (1.30)	Love et al.^91^	RRID:SCR_015687
GraphPad Prism 9	http://www.graphpad.com/	RRID:SCR_002798
WebGestaltR (0.4.4)	Liao et al.^92^	RRID:SCR_006786
Image Lab Software (6.1)	https://www.bio-rad.com/en-se/product/image-lab-software?ID=KRE6P5E8Z	RRID:SCR_014210

### Resource availability

#### Lead contact

Further information and requests for resources and reagents should be directed to and will be fulfilled by the lead contact Lena Eliasson (lena.eliasson@med.lu.se).

#### Materials availability

This study did not generate new unique reagents.

### Experimental model and subject details

#### Human pancreatic islets

Pancreatic islets from normal glucose tolerant (NGT) donors (HbA1c < 6%, n = 4) were received from the Human Tissue Lab EXODIAB/LUDC via the Nordic Network for Islet Transplantation (http://www.nordicislets.org). Islets were processed in accordance with procedures described in ethical permits issued by the Uppsala and Lund University Ethics committees.^88^ Islets were handpicked in cold Hank’s buffer with 1 mg/mL bovine serum albumin (BSA) before it was transferred to RPMI 1640 medium with 5 mM glucose supplemented with 10% Fetal Bovine Serum (FBS), 5 mL Penicillin/Streptomycin (10000 U/10 mg/mL) under a stereomicroscope. The islets were incubated in a humidified atmosphere with 5% CO^2^ at 37°C. Donor characteristics are included in Table S1.

#### EndoC-βH1 cells

EndoC-βH1 cells (EndoC-βH1 cells, Paris, France)^88^ were grown in Matrigel fibronectin-coated (100 μg/mL and 2 μg/mL, respectively, Sigma–Aldrich, Steinheim, Germany) culture vessels in DMEM (Thermo Fisher Scientific, Waltham, MA, USA) containing 5.6 mM glucose, 2% BSA fraction V (Roche Diagnostics, Mannheim, Germany), 10 mM nicotinamide (Merck Millipore, Darmstadt, Germany), 50 μM 2-mercaptoethanol, 5.5 μg/mL transferrin, 6.7 ng/mL sodium selenite (Sigma–Aldrich), 100 U/mL penicillin, and 100 μg/mL streptomycin (PAA Laboratories, Pasching, Austria). The cells were incubated in a humidified atmosphere with 5% CO^2^ at 37°C. The cells were tested for mycoplasma contamination on a regular basis.

### Method details

#### Dexamethasone treatment

Prior to functional and molecular experiments islets or EndoC-βH1 cells were incubated for indicated time-periods in cell culture medium containing dexamethasone (#D1756, Sigma-Aldrich) dissolved in DMSO (≥99.7%; 1:1000, #34869, Sigma-Aldrich).

#### Identification of potential direct gene targets of transcription factors in pancreatic islets/β-cells

To identify predicted GR gene targets RNA-seq data was integrated with publicly available high-confident Glucocorticoid Receptor ChIP-seq experimental data from the Gene Transcription Regulation Database (GTRD, GCR_HUMAN.A)^35^ as summarized in Figure 3A. First, differentially expressed (DE) genes (adjusted p-value <0.05) were associated with GR ChIP-peaks (or Glucocorticoid Responsive Elements, GREs) in a range of 150 kb of their transcription start site (TSS). Then genes with GREs that overlapped with open chromatin positions in human islets^93^ or EndoC-βH1 cells^94^ were considered as islet-specific. As human islet and EndoC-βH1 open chromatin data have different number of classes, and islet-specific factors show preference for binding islet open chromatin elements of different classes with distinct frequencies,^95^ selected EndoC-βH1 classes were corresponded to relevant human ones and were given a score according to those frequencies (Table S6). A comparison list between the different element composition of open chromatin regions in human islets and EndoC-βH1 cells is also provided (Table S6). The *Open Chromatin Score* for a gene is therefore defined as the sum of scores of all of its associated GREs and is based on the open chromatin classes they overlap. Next, enhancer-gene target associations were retrieved from GeneHancer (v.4.4), a comprehensive database of human enhancer elements, where gene-enhancer association scores derived from multi-sourced annotation of experimental data are included.^96^ The GREs of each DE gene were scored if they co-located with an enhancer that was associated with the corresponding gene. As a result, the *Enhancer-Target Score* for a gene is the sum of scores of all its associated GREs that overlap enhancers that target this specific gene. The last step includes the discovery of the GR binding site motif occurrences on the GREs of the genes. The enriched GR binding motif in all GREs was first captured with the MEME-ChIP tool (v. 5.0.2) with default parameters and the motif was scanned across the GREs associated with the DE genes using FIMO (motifs with matching p < 0.0001 were retained).^97^ DE gene’s *Open Chromatin Score*, *Enhancer-Target Score*, *GR motif number* were normalized with the feature scaling method, which rescales the range of values of each feature to a scale between 0 and 1 with the formula: normalized value = (value - min(value))/(max(value) - min(value)). Then the sum of the 3 normalized features produced the gene’s *Normalized Annotation Score*. Subsequently, the genes were associated with two ranks, depending on increasing “Differential Expression Change” (rank_DEC = 1 for the highest Differential Expression Change) and “Normalized Annotation Score” (rank_NAS = 1 for the highest Normalized Annotation Score) and the rank product was calculated: rank product = (rank_DEC/n) ∗ (rank_NAS/n), where n is the number of the DE genes. Genes with the lowest rank product, thus, have a higher probability of being directly targeted by GR.

A similar procedure for the detection of ZBTB16 gene targets was followed (Figure 7A), with the distinction that since differential gene expression data was not available, genes were ranked only according to their *Normalized Annotation Score*. After retrieving ZBTB16 ChIP-seq data from GTRD (ZBT16_HUMAN.A, ZBT16_HUMAN.B, ZBT16_HUMAN.C),^35^ all genes in the genome were associated with ZBTB16 ChIP-peaks within a 100 kb window of their TSS that overlapped with human islet open chromatin regions.

#### Single-cell transcriptomic expression data processing

Processed human islet single-cell transcriptomic expression data from 6 control donors (311 β-cells) was obtained from the GEO database (GEO: GSE153855). Provided gene symbols have been updated in R using biomaRt (v.2.50.2). Genes that were expressed in at least 1 cell and have an expression level of Reads Per Kilobase Million (RPKM) > 1 were considered expressed in β-cells and were used for downstream analysis.

#### Total RNA extraction and quantification by real-time quantitative PCR (qPCR)

Total RNA was extracted using the Qiagen miRNeasy isolation kit according to the manufacturer’s instructions (Qiagen, Hilden, Germany). RNA concentration and quality was determined using 2 μL on a NanoDrop Spectro-photometer (ND-1000, Thermo Fisher). RNAs that passed the quality control test were reversely transcribed to cDNA with the use of High Capacity cDNA Reverse Transcription kit according to the manufacturer’s instructions (Applied Biosystems, Waltham, MA, USA). qPCR was performed using TaqMan® gene expression assays (Applied Biosystems, CA, USA) for measuring the expression of ZBTB16 (Hs00232313_m1), GR (Hs00353740_m1), SGK1 (Hs00353740_m1), PDX-1 (Hs00236830_m1) and INS (Hs02741908_m1) in Applied Biosystems QuantStudio (TM) 7 Flex RT-PCR system under default cycling parameters with. HPRT1 (4333768F) and PPIA (4333763F) were both used as endogenous controls. The ΔΔCt method was applied for relative quantification and the recalibrated values (2^−ΔΔCt^) were presented as the fold-change with respect to control or untreated conditions.

#### RNA-sequencing processing and analysis

After total RNA extraction, RNA-seq libraries were prepared using the TruSeq Stranded Total RNA Library Prep with Ribo-Zero kit (#RS-122-2201, Illumina, CA, USA). Samples were loaded and sequenced using the Nextseq 500/550 High Output kit v2 (#FC-404-2002, Illumina, CA, USA) on an Illumina NextSeq 500 sequencer. After the quality of the generated sequence reads was assessed with FastQC v. 0.11.8,^89^ the reads were mapped to the human transcriptome (Gencode Release 27; genome assembly GRCh38) and quantified accordingly by Salmon v.0.14.0^90^ with parameters --incompatPrior 0.0 --validateMappings --gcBias --seqBias. Differential gene expression analysis was performed by DESeq2 v.1.30.0.^91^ Expressed genes included in subsequent analyses were considered those with normalized counts > 3 in at least 80% of the samples in each of the treated/control group and significantly differentially expressed genes were considered those with adjusted p-value <0.05.

#### Functional annotation

Functional annotation of gene sets was performed with over-representation analysis using the R package WebGestaltR (v. 0.4.4), based on the WebGestalt database.^92^ Gene sets were searched against terms/pathways from the following functional categories: Gene Ontology (GO), Reactome pathways, KEGG pathways and the Molecular Signatures Database (MSigDB) hallmark gene set collection. Significantly enriched functional annotation terms included those with an adjusted for multiple testing FDR < 0.05 (Benjamini–Hochberg method). Selected terms/pathways were displayed in the figures as relevant/representative of the full list.

#### Western blotting analysis

After treating EndoC-βH1 cells with dexamethasone, the cells were lysed and protein concentration was determined with the BCA protein assay kit (Pierce, Rockford, IL, USA). Protein (15 μg homogenate) was separated by 4–15% TGX Stain-Free gels (Bio-Rad, Hercules, CA, USA). Then, the gels were activated with UV light for 1 min to visualize total protein on the blotted LF PVDF membrane (Bio-Rad). The protein was transferred to PVDF membranes, then blocked with 5% milk and 1% BSA in buffer consisting of 150 mM NaCl, 20 mM Tris-HCl, pH 7.5, and 0.1% (v/w) Tween for 1 h. The membranes were individually probed with an antibody against ZBTB16 (1:1000, #39784, Cell Signaling Technology, Danvers, MA, USA). The primary antibody was detected with a horseradish peroxidase conjugated goat anti-rabbit IgG, HRP-linked antibody (1:10 000, #7074, Cell Signaling Technology, Danvers, MA, USA). Protein was detected with Clarity Western ECL Substrate and Bio-Rad ChemiDoc MP Imaging System (Bio-Rad Laboratories) and quantified with Image Lab 6.1 software (Bio-Rad Laboratories), after normalizing the intensity of each protein band to that of the total protein bands in the lane.

#### ChIP assay

For ChIP–PCR experiments, 20 × 10^6^ EndoC-βH1 cells were treated with 100 nM dexamethasone or DMSO (Control) for 24h. Fixation, enzymatic shearing, and immunoprecipitation were performed with the ChIP-IT® Express Enzymatic kit according to the manufacturer’s instructions (Active Motif, Carlsbad, CA, USA). After shearing, part of the chromatin solution was stored and served as PCR control (Input DNA), while the rest was immunoprecipitated with 10 μl of anti-GR antibody (1:100, #12041, Cell Signaling Technology, Danvers, MA, USA). After purification of the antibody-bound complexes, PCR was performed in the purified immunoprecipitated DNA for specific genomic positions (primers in Table S7). PCR products were separated on a 2.5% agarose gel at 100V and visualized by UV-illumination following staining with GelRed (Biotium, CA, USA) with Image Lab 6.1 software (Bio-Rad Laboratories).

#### Gene overexpression

EndoC-βH1 cells were seeded in a 48-well plate (180,000 cells/well) containing 150 μL medium without antibiotics (penicillin/streptomycin) a day before transfection. The cells were either transfected with a plasmid expressing ZBTB16 (ZBTB16_OHu04229C_pcDNA3.1(+)-C-HA, #SC1200, Genescript, New Jersey, U.S.A) or a control plasmid pcDNA3.1(+) (Genescript, New Jersey, U.S.A) using Lipofectamine LTX (#15338-100, Thermo Fisher). In the end, a final transfection volume of 50 μL/well contained 0.25 μg of plasmid in Opti-MEM reduced serum media and 0.5 μL of Lipofectamine LTX. The cells reached 90–100% confluence ≈72 h post-transfection and were assayed for insulin secretion and used for protein and RNA extraction as described.

#### Gene silencing

EndoC-βH1 cells were seeded in a 48-well plate (180,000 cells/well) containing 150 μL medium without antibiotics a day before transfection. After 24 h the cells were transfected with Silencer Select Pre-Designed siRNA against ZBTB16 (n341241; #4390771, Thermo Fisher) or Silencer Select Negative Control No. 2 siRNA (#AM4613, Thermo Fisher). A final transfection volume of 50 μL per well contained 25 nmol/L siRNA in Opti-MEM reduced serum media and 0.5 μL Lipofectamine RNAiMAX (#13778075, Thermo Fisher). A second transfection was performed 24 h after the first transfection and 4–6 h before 100 nM dexamethasone treatment. The cells reached 90–100% confluence ≈48 h after the second transfection and were assayed for insulin secretion and used for protein and RNA extraction.

#### Insulin secretion assay

Confluent EndoC-βH1 plates were washed twice with 1 mL pre-warmed secretion assay buffer (SAB), pH 7.2 (1.16 mM MgSO4, 4.7 mM KCl, 1.2 mM KH2PO4, 114 mM NaCl, 2.5 mM CaCl2, 25.5 mM NaHCO3, 20 mM HEPES, and 0.2% bovine serum albumin) containing 1 mM glucose, before they were incubated with 0.5 mL SAB containing 1 mM glucose for 2 h. The cells were then stimulated in low-glucose condition with 0.25 mL SAB containing 1 mM glucose supplemented with 500 μM IBMX (I5879-1G, Sigma-Aldrich, Steinheim, Germany) or in high-glucose condition with 20 mM glucose supplemented with 500 μM IBMX for 1h at 37°C. Insulin secretion and content were measured using Mercodia Insulin ELISA (human, #10-1113-01, Uppsala, Sweden). Protein from each well was extracted using 100 μL RIPA buffer: 0.1% SDS, 150 nM NaCl, 1% Triton X-100, 50 mM Tris-Cl, pH 8, and EDTA-free protease inhibitor (Roche, Branchburg, NJ, USA) and quantified with BCA assay (Pierce, Rockford, IL, USA). Insulin secretion and content values were normalized to the total protein content of the corresponding wells.

#### Cell viability/proliferation assay

Cell Viability was assessed with MTS assay (CellTiter 96® AQueous One Solution Cell Proliferation Assay, #G3582, Promega, U.S.A). EndoC-βH1 cells were seeded in a 96-well plate (90,000 cells/well) containing 75 μL medium without antibiotics and were treated with a final transfection volume of 25 μL with either a plasmid expressing ZBTB16/control plasmid (*ZBTB16* overexpression) or a Silencer Select ZBTB16 siRNA/Silencer Select Negative Control No. 2 siRNA (*ZBTB16* knockdown) as mentioned above. On the day of the assay 20 μL CellTiter 96® AQueous One Solution Reagent were added directly to the culture medium in each plate well followed by incubation at 37°C for 2 h in a humidified, 5% CO_2_ atmosphere. Finally, absorbance was measured at 490 nm and was normalized by the total protein content of each well.

#### Mitochondrial oxygen consumption rate measurements

EndoC-βH1 cells were seeded in 24-well cell plates (Agilent Technologies, Santa Clara, CA, USA, cat. no.: 102340-100) intended for use in the Seahorse XFe24 analyzer system (Agilent Technologies, USA). In each well, 60,000 cells were seeded in 75 μL antibiotic free medium. One day post-seeding, overexpression of ZBTB16 or a control plasmid was performed as described in section gene overexpression with adjustment for lower volumes. Two days post-seeding, the medium was replaced with medium containing dexamethasone at a 100 nM concentration or control DMSO. Three days post-seeding, the medium was replaced with starvation medium containing glucose at a 2.8 mM concentration. Four days post-seeding, ≈72 h post-transfection of plasmids, the cells reached a confluence of 90–100%. Cells were washed with PBS and pre-incubated 2 h in a modified secretion assay buffer (exclusion of NaHCO3 and bovine serum albumin) as described in section insulin secretion assay. Mitochondrial oxygen consumption rate (OCR) was measured in the Seahorse XFe24 analyzer at baseline of 1 mM glucose, after stepwise injections of 10 mM pyruvate, 5 μg/mL oligomycin, 4 μM carbonyl cyanide p-trifluoromethoxy-phenylhydrazone (FCCP) and 1 μM rotenone. OCR in each well was analyzed using the specified online tool for the analyzer system (URL: seahorseanalytics.agilent.com) and normalized to total protein in each well quantified with BCA assay (Pierce, Rockford, IL, USA).

### Quantification and statistical analysis

Statistical analysis of experimental results was performed with GraphPad Prism version 9 (GraphPad Software, Inc., La Jolla, CA, USA). The distribution of the data in each experimental setup was assessed before conducting further analyses. For comparing qPCR results the Wilcoxon matched pairs signed rank test was used. For other comparisons repeated measures ANOVA (1-, 2- or 3-way) was performed. Post-hoc pairwise comparisons included Tukey multiple comparisons for dose- and time-dependent dexamethasone treatment experiments and paired Student t-tests for insulin secretion and insulin content experiments. Data are presented as mean ± SEM. Statistical significance is represented with asterisks as: ∗p < 0.05, ∗∗p < 0.01, ∗∗∗p < 0.001.

## Data Availability

• RNA-sequencing data generated in the current study have been submitted to GEO database and are publicly available. Accession number is listed in the key resources table. • This paper does not report original code. • Any additional information required to reanalyze the data reported in this paper is available from the lead contact upon request.

## Supplemental information

Document S1. Figures S1–S4 and Tables S1 and S7

Table S2. Differentially expressed genes in human islets and EndoC-βH1 cells, related to Figure 1

Table S3. Functional Annotation of DE genes, related to Figure 1

Table S4. ZBTB16 predicted gene targets in Human Islets and Beta Cells, related to Figure 7

Table S5. Functional Annotation of ZBTB16 predicted gene targets, related to Figure 7

Table S6. Open Chromatin Composition in Human Islets and EndoC-βH1 cells, related to Figures 3 and 7
