# Supplementary material for: Glucocorticoid-mediated induction of ZBTB16 affects insulin secretion in human islets and EndoC-βH1 β-cells
Source: iScience. 2023 Apr 1;26(5):106555. doi: 10.1016/j.isci.2023.106555 (PMC10214295; doi:10.1016/j.isci.2023.106555)
Supplement: Document S1. Figures S1–S4 and Tables S1 and S7 [file mmc1.pdf]

iScience, Volume *26*

Supplemental information

Glucocorticoid-mediated induction of ZBTB16 affects insulin secretion in human islets and EndoC-βH1 β-cells

Alexandros Karagiannopoulos, Efraim Westholm, Jones K. Ofori, Elaine Cowan, Jonathan L.S. Esguerra, and Lena Eliasson

Figure S1. Glucocorticoid Receptor ChIP assay followed by PCR on the predicted Glucocorticoid Receptor Elements (GREs) on the *ZBTB16* gene, related to Figure 4C. For each GRE, the gel band intensity of the GR ChIP band in each replicate was normalized to that of the input band for each condition (Ctrl = control ChIP/control Input, Dexa = Dexa ChIP/Dexa Input) using the ImageLab software (version 6.1, Bio-Rad Laboratories) and the Fold Enrichment of the normalized signal after treatment was measured with respect to control (Dexa/Ctrl) (n=3). The red dashed line represents the baseline where no difference in GR precipitation is observed (Fold enrichment = 1).

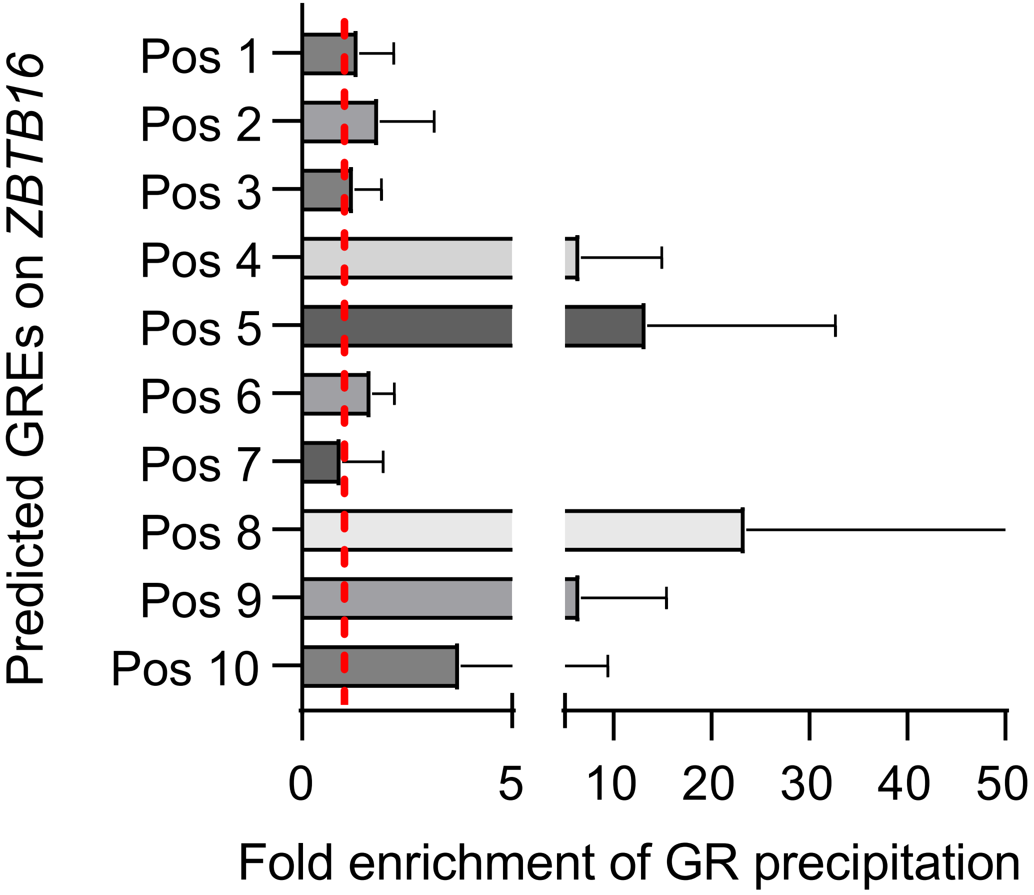

Figure S2. Insulin secretion (% insulin content) measurements, related to Figures 5D, 5E, 6C, 6J. (A) Insulin secretion of EndoC-βH1 cells upon treatment with dexamethasone at different time points (n=6 biological replicates), complementary to Figures 5D,E. (B) Insulin secretion of EndoC-βH1 cells after ZBTB16 overexpression treated with dexamethasone (n=5 biological replicates),complementary to Figure 6C. (C) Insulin secretion of EndoC-βH1 cells after ZBTB16 induction suppression and dexamethasone treatment (n=6 biological replicates), complementary to Figure 6J. Multiple Wilcoxon tests were performed to assess the significance level of all comparisons. Low glucose = 1 mM, high glucose = 20 mM, Ctrl = Control (DMSO), Dexa = 100nM Dexamethasone. Data are presented as mean ± SEM; *p < 0.05.

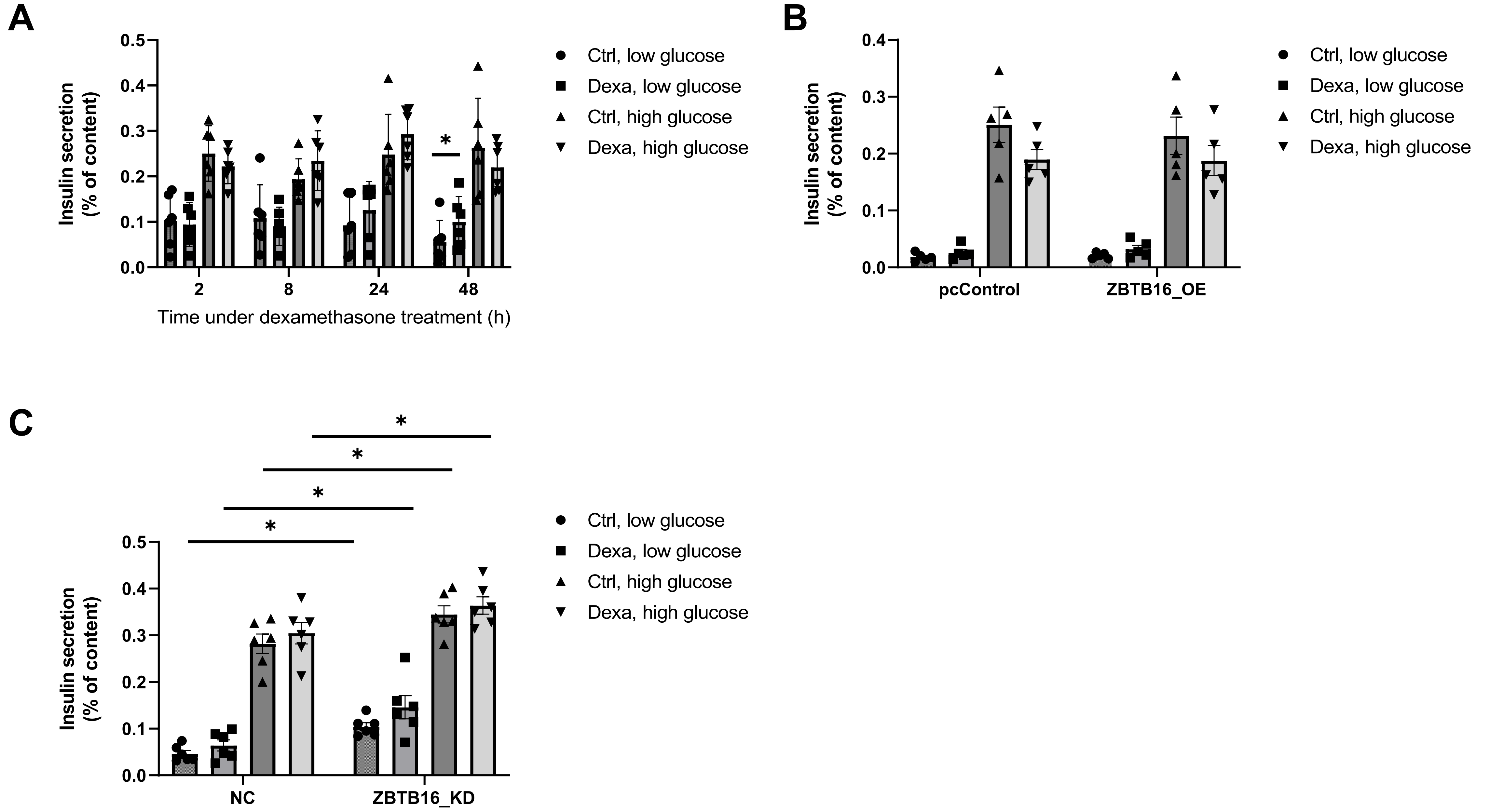

Figure S3. **Cell viability of EndoC cells after dexamethasone treatment during overexpression/induction suppression of *ZBTB16,* related to Figure 7.** Cell viability of EndoC cells, as measured with MTS assay, after treatment with 100nM dexamethasone for 48h (n=4 biological replicates) relative to the control condition. *ZBTB16* induction suppression (left) and overexpression (right) for 72h with the respective controls are shown in the barplots. Paiwise comparisons were performed with repeated measures 2-way ANOVA for each condition followed by pairwise comparisons with Tukey's multiple comparison test. Ctrl = Control (DMSO), Dexa = Dexamethasone, pcControl = Control plasmid pcDNA3.1, ZBTB16_OE = ZBTB16 overexpression, NC = Negative Control, ZBTB16_KD = ZBTB16 knockdown. Data are presented as mean ± SEM; **p < 0.01, ***p < 0.001.

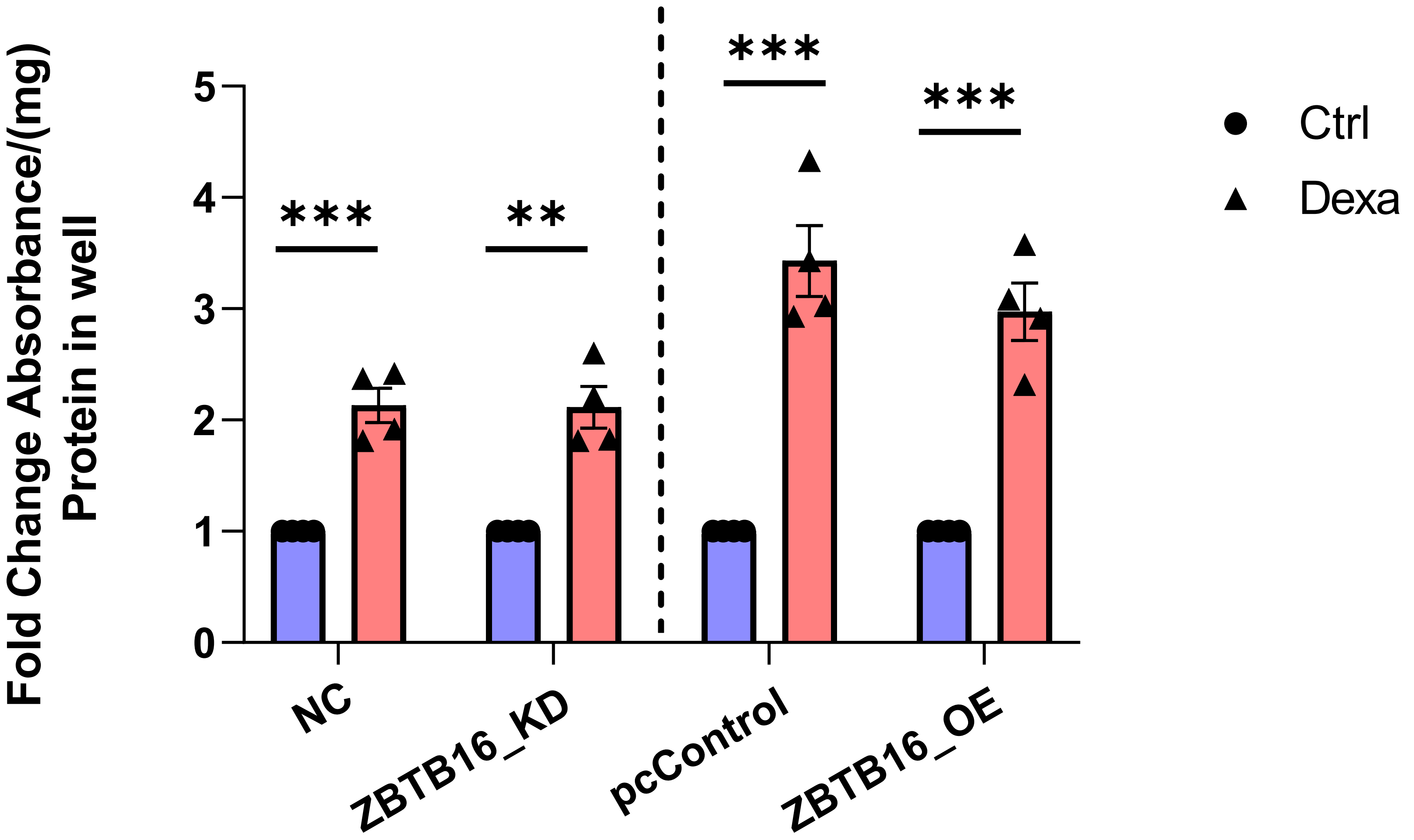

Figure S4. Full-length Western blot images, related to Figure 3D. EndoC-βH1 cells (n=6 biological eplicates) treated with DMSO (Ctrl) or 2μM dexamethasone (Dexa).

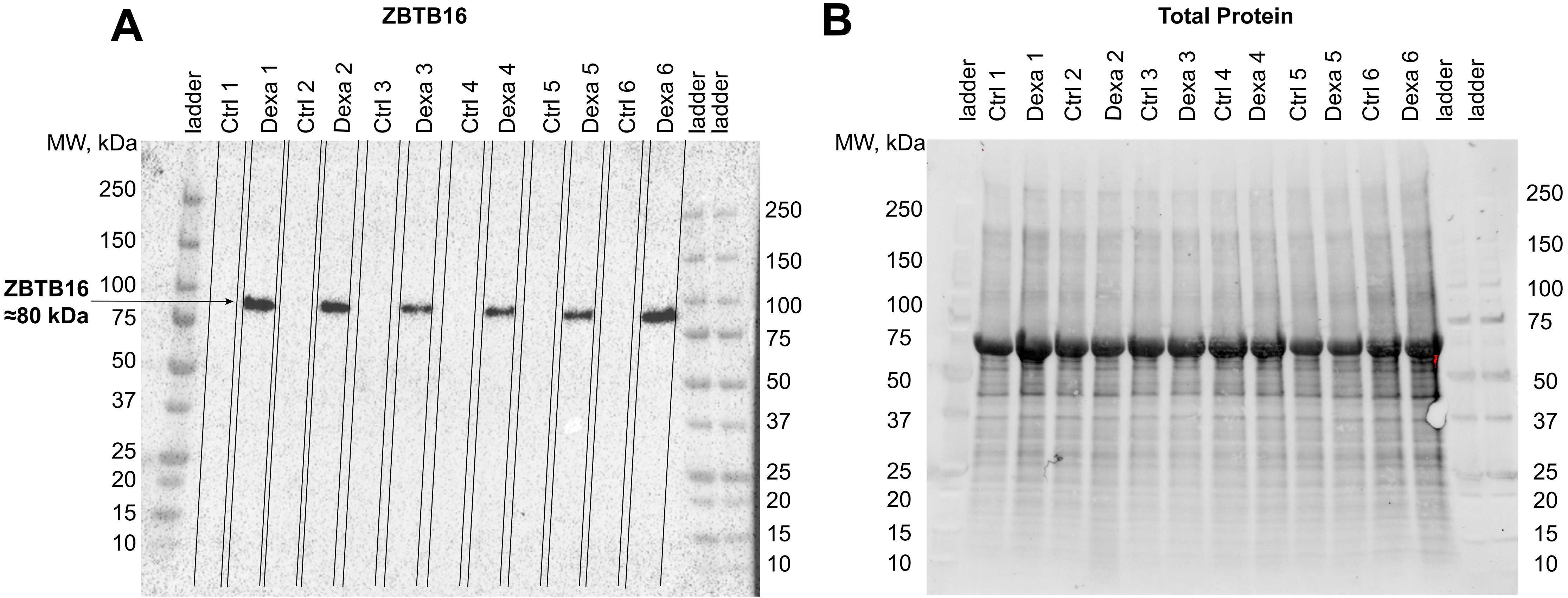

Table S1. Human Islet Donor Characteristics, related to Figure 1

Donor ID	Gender	Age, yr	BMI	HbA1c, %	HbA1c (mmol/mol)
1	Male	66	24.3	5.7	39
2	Male	67	26.1	5.6	38
3	Female	81	22.8	5.7	39
4	Male	43	30.9	5.8	40

Table S7. Primers for Glucocortcoid Receptor (GR) binding sites on *ZBTB16* gene, related to Figure 4

Position number	Position ID/Coords (hg38)	Forward (5'->3')	Reverse (5'->3')	Size (bp)
1	*chr11_114159912_114160415*	GAGCATTGCCTGTGTTTGTTAT	GGAATTCAATGCGGCCTATTC	211
2	*chr11_114162764_114163180*	GTGATGCGGATGCAGAGAGT	CAGTGACGACACCACTCTGG	143
3	*chr11_114179131_114179656*	AGTGTGCTGTTCTCCGTCTG	ATCTGCACGCCTGCTTACAT	166
4	*chr11_114098946_114099330*	CTCAGCCCTGTTCTCCTTTC	CCCAGTAAGATCACGGTTGTT	218
5	*chr11_114080268_114080696*	CATCCACTGAGCCCAAGATT	TAGCACAACCTGGCCTTAAATA	277
6	*chr11_114197742_114198458*	GTATCGGGGAGGACAGGACA	AAGTGCCTATGACGTTCCGC	204
7	*chr11_114173033_114173448*	AAACACCAAAGGCAAGCCAAT	TACTCCCCAACACCCATTCAG	141
8	*chr11_114124563_114124916*	GCATCTCTTGGCCATATCCCT	TGCGTCCAACTGACAATGAGA	153
9	*chr11_114139420_114140069*	CCTCCTGGCAAGTGACCAAA	TGCCGTCCTTCCTATCCTGA	180
10	*chr11_114180474_114180830*	CCTAGGGGACAGACAGCTTG	AGTCTGTGCCCACTAAATGCT	122
